# Persistent Body Mass Index Profiles During Adolescence: Associations With Brain Maturation and DNA Methylation

**DOI:** 10.1016/j.bpsgos.2026.100820

**Published:** 2026-09-01

**Authors:** Xinyang Yu, Shitong Xiang, Tianye Jia, Zuo Zhang, Yan Sun, Tobias Banaschewski, Gareth J. Barker, Arun L.W. Bokde, Rüdiger Brühl, Hugh Garavan, Penny Gowland, Antoine Grigis, Andreas Heinz, Hervé Lemaître, Jean-Luc Martinot, Marie-Laure Paillère Martinot, Eric Artiges, Frauke Nees, Dimitri Papadopoulos Orfanos, Luise Poustka, Michael N. Smolka, Sarah Hohmann, Nathalie Holz, Nilakshi Vaidya, Henrik Walter, Robert Whelan, Ulrike Schmidt, Gunter Schumann, Sylvane Desrivières, IMAGEN Consortium, Tobias Banaschewski, Gareth J. Barker, Arun L.W. Bokde, Rüdiger Brühl, Hugh Garavan, Penny Gowland, Antoine Grigis, Andreas Heinz, Hervé Lemaître, Jean-Luc Martinot, Marie-Laure Paillère Martinot, Eric Artiges, Frauke Nees, Dimitri Papadopoulos Orfanos, Luise Poustka, Michael N. Smolka, Sarah Hohmann, Nathalie Holz, Nilakshi Vaidya, Henrik Walter, Robert Whelan, Gunter Schumann, Sylvane Desrivières

**Affiliations:** a Social, Genetic and Developmental Psychiatry Centre, Institute of Psychiatry, Psychology and Neuroscience, King’s College London, London, United Kingdom; b Institute of Science and Technology for Brain-Inspired Intelligence, Fudan University, Shanghai, China; c Key Laboratory of Computational Neuroscience and Brain-Inspired Intelligence, Fudan University, Ministry of Education, Shanghai, China; d Centre for Population Neuroscience and Stratified Medicine, Institute of Science and Technology for Brain-Inspired Intelligence, Fudan University, Shanghai, China; e National Institute on Drug Dependence, Peking University, Beijing, China; f Department of Child and Adolescent Psychiatry and Psychotherapy, Central Institute of Mental Health, Medical Faculty Mannheim, Heidelberg University, Mannheim, Germany; g Department of Neuroimaging, Institute of Psychiatry, Psychology and Neuroscience, King’s College London, London, United Kingdom; h Discipline of Psychiatry, School of Medicine and Trinity College Institute of Neuroscience, Trinity College Dublin, Dublin, Ireland; i Physikalisch-Technische Bundesanstalt, Braunschweig and Berlin, Germany; j Departments of Psychiatry and Psychology, University of Vermont, Burlington, Vermont; k Sir Peter Mansfield Imaging Centre School of Physics and Astronomy, University of Nottingham, Nottingham, United Kingdom; l NeuroSpin, Commissariat à l'énergie Atomique et aux Énergies Alternatives, Université Paris-Saclay, Gif-sur-Yvette, France; m Department of Psychiatry and Psychotherapy, Campus Charité Mitte, Charité Universitätsmedizin Berlin, corporate member of Freie Universität Berlin, Humboldt-Universität zu Berlin, and Berlin Institute of Health, Berlin, Germany; n Institut des Maladies Neurodégénératives, Unité Mixte de Recherche 5293, Centre National de la Recherche Scientifique, Commissariat à l'énergie Atomique et aux Énergies Alternatives, Université de Bordeaux, Bordeaux, France; o Institut National de la Santé et de la Recherche Médicale, U1299 Developmental Trajectories & Psychiatry, Université Paris-Saclay, Université Paris Cité, École Normale Supérieure Paris-Saclay, Centre National de la Recherche Scientifique, Centre Borelli Unité Mixte de Recherche 9010, Gif-sur-Yvette, France; p Department of Child and Adolescent Psychiatry, L'Assistance Publique-Hôpitaux de Paris, Sorbonne Université, Pitié-Salpêtrière Hospital, Paris, France; q Institute of Cognitive and Clinical Neuroscience, Central Institute of Mental Health, Medical Faculty Mannheim, Heidelberg University, Mannheim, Germany; r Institute of Medical Psychology and Medical Sociology, University Medical Center Schleswig Holstein, Kiel University, Kiel, Germany; s Department of Child and Adolescent Psychiatry and Psychotherapy, Center for Psychosocial Medicine, University Hospital Heidelberg, Heidelberg, Germany; t Department of Psychiatry and Psychotherapy, Technische Universität Dresden, Dresden, Germany; u Department of Child and Adolescent Psychiatry, Psychotherapy and Psychosomatics, University Medical Center Hamburg-Eppendorf, Hamburg, Germany; v Centre for Population Neuroscience and Stratified Medicine, Department of Psychiatry and Psychotherapy, Charité Universitätsmedizin Berlin, Berlin, Germany; w School of Psychology and Global Brain Health Institute, Trinity College Dublin, Dublin, Ireland; x Department of Psychological Medicine, Centre for Research in Eating and Weight Disorders, Institute of Psychiatry, Psychology and Neuroscience, King’s College London, London, United Kingdom; y South London and Maudsley National Health Service Foundation Trust, London, United Kingdom; z Centre for Population Neuroscience and Stratified Medicine, Department of Psychiatry and Neuroscience, Charité Universitätsmedizin Berlin, Berlin, Germany; aa Centre for Population Neuroscience and Precision Medicine, Institute of Science and Technology for Brain-Inspired Intelligence, Fudan University, Shanghai, China

**Keywords:** Adolescence, Body mass index, Cortical maturation, Disordered eating behaviors, DNA methylation, Structural neuroimaging

## Abstract

**Background:**

Adolescence is a critical period for the emergence of obesity and disordered eating behaviors, yet the biological mechanisms underlying persistent differences in body mass index (BMI) development remain poorly understood. We investigated whether longitudinal BMI profiles are associated with behavioral, neurodevelopmental, and epigenetic variation during adolescence.

**Methods:**

Participants from the IMAGEN cohort (*N* = 1296) were followed from ages 14 to 23 years. Latent profile analysis identified longitudinal BMI profiles. Disordered eating behaviors were repeatedly assessed across adolescence. Structural magnetic resonance images acquired at ages 14 and 23 characterized longitudinal changes in gray matter volume (GMV), cortical thickness (CT), and sulcal depth. Genome-wide DNA methylation (DNAm) was measured in whole blood at age 14. We conducted epigenome-wide association studies (EWASs) and functional enrichment analyses and evaluated a DNAm-based BMI risk score (bmiMRS).

**Results:**

Two distinct BMI profiles were identified: a persistently overweight group and a persistently normal-weight group. Adolescents with persistent overweight showed progressively increasing BMI; greater eating-, weight-, and shape-related concerns; and more frequent dieting and binge-eating behaviors. They also exhibited atypical cortical maturation, characterized by attenuated cortical thinning, accelerated reductions in sulcal depth, and reduced gray matter volume loss within frontal and temporal association cortices. EWAS identified 29 BMI-associated CpGs (Bonferroni-corrected *p* < 1.3 × 10^−7^), enriched for genes involved in adipogenesis, immune and inflammatory signaling, type 2 diabetes, and brain-related biological processes. The bmiMRS predicted BMI, cumulative BMI across adolescence, and membership of the persistently overweight group and remained significantly associated with BMI in an independent cohort. Longitudinal analyses further suggested reciprocal associations between BMI and brain maturation, with higher BMI in early adolescence predicting subsequent regional GMV changes, whereas lower baseline CT predicted greater BMI increases independently of baseline BMI.

**Conclusions:**

Persistent adolescent overweight is associated with distinct behavioral, neurodevelopmental, and peripheral epigenetic signatures. Integrating longitudinal neuroimaging and DNAm identifies molecular biomarkers and brain developmental features associated with sustained BMI elevation, providing a framework for earlier risk stratification and informing biologically grounded approaches to obesity prevention.

## Plain Language Summary

Adolescence is a critical period for the development of overweight and obesity, but the biological factors linked to long-term weight gain are not fully understood. We followed 1296 young people from ages 14 to 23 years and combined information on body weight, eating behaviors, brain scans, and DNA methylation—a molecular marker that can reflect genetic and environmental influences. Adolescents with persistent overweight showed greater eating-related concerns, differences in brain maturation, and distinct DNA methylation patterns linked to metabolism and inflammation. These findings may help identify young people at risk and support earlier and more targeted approaches to obesity prevention.

Overweight and obesity affect more than 1.5 billion people worldwide (1,2) and represent a major global public health challenge (3). Body mass index (BMI) remains the most widely used indicator of adiposity, and a high BMI is a major risk factor for cardiovascular and metabolic diseases (4,5).

Disordered eating and weight-related concerns are particularly prevalent among adolescents with overweight and obesity (6,7). Longitudinal studies have shown that these behaviors and concerns can lead to substantial weight gain over 10 years (8) and are associated with adverse mental health outcomes, such as suicidal ideation (9) and mental distress (10). These findings highlight the importance of assessing adolescents’ weight-related concerns and disordered eating behaviors (DEBs) and determining whether such concerns are precursors to, or consequences of, sustained overweight status. Longitudinal designs are particularly suited to exploring these temporal relationships.

BMI reflects interacting lifestyle, environmental, and genetic influences (11,12). Genome-wide association study variants explain approximately 22% (SE = 3.7%) of BMI variation (13), substantially less than twin-based estimates of 31% to 90% (14), implicating additional gene-environment and epigenetic mechanisms (15). DNA methylation (DNAm) captures genetic and environmental influences (16, 17, 18, 19, 20, 21), yet BMI epigenome-wide association study (EWAS) findings have been inconsistently replicated (22,23). Few studies span adolescence to early adulthood (24, 25, 26) or examine longitudinal BMI-related methylation profiles.

Neuroimaging studies of obesity and high BMI have reported lower gray matter volume (GMV) in regions involved in behavioral control, reward processing, emotional regulation, homeostasis, motor control, and mesolimbic functioning (27, 28, 29, 30). Large-scale studies, such as the UK Biobank (*N* > 9000), have also linked higher BMI to smaller subcortical structures (27). However, most evidence is cross-sectional and adult based (29,31), limiting the understanding of BMI-related neurodevelopment during adolescence.

Our recent longitudinal study linked genetic predisposition to higher BMI, behavioral problems, protracted brain maturation, and eating-disorder symptoms (32). Distinct regional volume changes mediated the associations of BMI polygenic scores and externalizing problems with eating behaviors, highlighting the value of multimodal longitudinal assessment.

Here, in this study, we integrated longitudinal behavioral, neuroimaging, and epigenetic data from adolescents followed from ages 14 to 23 years. We identified longitudinal BMI profiles and tested differences in disordered eating behaviors, concerns, and maturation of GMV, cortical thickness (CT), and sulcal depth (SD). We also conducted a BMI EWAS at age 14 and functional enrichment analyses and evaluated a DNAm-derived BMI risk score (bmiMRS). Finally, we examined longitudinal relationships among early BMI, brain maturation, and DNAm to better understand how behavioral, neurodevelopmental, and epigenetic factors jointly relate to adolescent BMI development (Figure S1).

## Methods and Materials

### Participants

Participants were enrolled in the IMAGEN cohort, a multicenter longitudinal adolescent study conducted at 8 European sites (33). The study protocol was approved by local ethics research committees at each site, and signed informed consent was obtained from the participants and their parents or guardians.

### Phenotypical Assessments

BMI was calculated as weight divided by height squared. Categories for ages 14 to 19 used Centers for Disease Control and Prevention sex- and age-specific percentiles, whereas categories at age 23 used adult thresholds (Supplement). BMI increase was captured by the area under the curve (AUC) across ages 14, 19, and 23 using the trapezoidal method, which integrates BMI values across the 3 assessments weighted by the time interval between assessments.

DEBs (such as dieting, binge eating, and purging) and eating-, weight-, and shape-related concerns were derived from participant reports on the Development and Well-Being Assessment (34) at ages 14, 16, 19, and 23 (Supplement).

### Structural Magnetic Resonance Imaging Data

Structural magnetic resonance imaging (MRI) data were acquired at ages 14 and 23 across 8 IMAGEN recruitment sites using 3T scanners from multiple manufacturers (Siemens, Philips, General Electric, and Bruker). Further details of the MRI preprocessing procedures are provided in (35) and in the Supplement.

### DNA Methylation

Whole-blood DNAm at age 14 was measured using the Illumina Infinium HumanMethylation450 BeadChip (450K array; Illumina). Data underwent quality control and stratified quantile normalization using minfi, leaving 372,582 CpGs from 1338 participants (Supplement). Several potential confounding factors were identified, including 1) the first 4 principal components (PCs) of methylation levels across autosomal probes, 2) hybridization wave, and 3) the first 2 PCs derived from estimated cell-type proportions. To further account for population stratification, the first 4 multidimensional scaling (MDS) components derived from genome-wide genotype data were included as covariates. Genotyping and ancestry quality control followed published procedures (32); only participants of European ancestry were included.

### Statistical Analyses

Except for latent profile analysis (LPA), analyses used R (version 4.3.2). Behavioral models adjusted for sex and recruitment site. Neuroimaging models additionally adjusted for time-appropriate total intracranial volume (TIV); longitudinal GMV models used change in TIV. DNAm models adjusted for sex, site, 4 genetic multidimensional scaling MDS components, 2 cell-composition PCs, 4 DNAm PCs, and hybridization wave.

#### Identification of Groups of Distinct BMI Profiles by LPA

Longitudinal LPA was conducted using Mplus version 8.8 to identify distinct BMI trajectory groups from ages 14 to 23. This approach classifies individuals into subgroups based on shared developmental patterns of BMI (e.g., persistently high vs. normal profiles). Models were fitted starting from a single-class solution up to 4-class solutions, and fit criteria were compared to determine the optimal model (Table S1 and Supplement).

#### Group Differences in DEBs, Eating Disorder–Related Concerns, and BMI

Group differences in DEBs, eating disorder (ED)–related concerns, and BMI were assessed separately at each age (i.e., 14, 16, 19, and 23 years) using logistic regression adjusted for sex and recruitment site. Odds ratios (ORs) and 95% CIs were reported, with Bonferroni correction across phenotypes at each age (i.e., 0.05/7 phenotypes or 0.05/6 at age 16, where BMI data were unavailable).

#### Group Differences in ED-Related Concern Trajectories

Latent growth curve models were conducted using the lavaan R package (version 0.6-17) to estimate intercepts and linear slopes for eating-, weight-, and shape-related concerns at ages 14, 16, 19, and 23. Time-invariant covariates included sex, recruitment site, and latent BMI trajectory group (persistent overweight vs. normal BMI). Missing data in ED-related questionnaire measures primarily resulted from participant attrition across follow-up assessments and from incomplete questionnaire responses at individual waves. These data were handled using full-information maximum likelihood estimation under the assumption of missing at random. Group differences in intercept and slope parameters were then tested to determine whether ED-related concerns differed across BMI trajectory groups over time.

#### Neuroimaging Analyses

Whole-brain voxel-based morphometry (VBM) used SPM12 general linear models. Two-sample *t* tests examined cross-sectional GMV differences between groups at ages 14 and 23 and longitudinal GMV differences over this interval, with the covariates specified above. Cluster-wise familywise error (FWE) correction was applied at a *p*_FWE_ < .05, with a cluster-forming threshold of *p* < .001; significant clusters were labeled using the Automated Anatomical Labeling 3 atlas.

Longitudinal CT and SD from 68 Desikan-Killiany regions were analyzed using linear mixed models with age, group, age-by-group interaction, and sex as fixed effects, and participants nested within site as a random effect. For both measures, Bonferroni correction was applied (*p*_Bonferroni_ = .05/68 regions of interest = 7.35 × 10^−4^).

#### EWAS of BMI, Differentially Methylated Regions, and Sensitivity Analysis

At age 14, CpG-wise linear regressions were used to test associations between DNAm beta values and BMI using the specified DNAm covariates. Epigenome-wide significance was *p* < 1.3 × 10^−7^ (0.05/372,582). BMI-associated differentially methylated regions (DMRs) were identified using comb-p (36), with a 500-bp autocorrelation window, seed *p* value < .01, Šidák-corrected *p* < .05, and at least 2 CpGs per region. Sensitivity analyses additionally adjusted for lifetime smoking or negative life-event frequency (37,38).

#### Functional Enrichment Analyses

Enrichment analyses were conducted using genes annotated to false discovery rate (FDR)–significant CpGs identified in the primary EWAS (*p*_FDR_ < .05; 157 CpGs). Separate analyses were performed for CpGs positively associated with BMI (*n* = 54) and CpGs negatively associated with BMI (*n* = 103).

Gene annotations were performed using GREAT’s default basal-plus-extension model on hg19, with proximal regions spanning from 5 kb upstream to 1 kb downstream of transcription start sites and with a distal extension to the nearest gene.

Differential tissue expression, cell-type signatures, and pathway enrichment analyses were performed using the GENE2FUNC module in FUMA (39,40). All genes available in FUMA (*N* = 57,241) were used as the background gene set.

Chromatin state enrichment used the core 15-state ChromHMM model across brain, blood, and T-cell reference tissues. For each chromatin state, the overlap between BMI-associated CpGs and chromatin state annotations was compared with the overlap observed among all CpGs tested in the EWAS. Enrichment ratios were calculated as the observed proportion divided by the expected proportion. Statistical significance was assessed using Fisher’s exact tests with FDR correction.

#### DNAm-Based BMI Risk Score

Methylation risk scores for BMI, bmiMRSs, were constructed using a least absolute shrinkage and selection operator (LASSO)–penalized regression model with 10-fold cross-validation, following methods described previously (38). Before model fitting, DNAm beta values and BMI were residualized for the same covariates included in the EWAS analyses.

#### bmiMRS: In-Sample MRS (Internal Cross-Validation)

LASSO regression was implemented using the biglasso package in R, with residualized BMI as the outcome and residualized DNAm values as predictors. The penalty parameter (λ) was selected using 10-fold cross-validation based on the minimum cross-validated mean squared error (MSE). CpGs with nonzero coefficients in the final model were retained, and their coefficients were used as weights. For each fold, the model was trained on 90% of the participants and applied to the remaining 10%, generating cross-validated predictions for all individuals. The final LASSO model retained 91 CpGs with nonzero coefficients (Table S12). These out-of-sample predicted values for CpGs were used as the in-sample bmiMRSs. Predictive performance was assessed using the Pearson correlation coefficient, out-of-sample *R*^2^, and MSE between observed and predicted BMI residuals. This cross-validated bmiMRS was subsequently tested for associations with BMI trajectory group membership, BMI AUC across adolescence and emerging adulthood, and structural brain development measures identified in the neuroimaging analyses.

#### bmiMRS: External Validation in the STRATIFY Sample

The 91 CpGs and IMAGEN-derived weights were evaluated in the STRATIFY (Brain Network-Based Stratification of Reinforcement-Related Disorders) sample (*N* = 359; mean age = 22.79 ± 2.60 years). The bmiMRS was calculated as the weighted sum of DNAm beta values across these CpGs. Associations between the bmiMRS—calculated as the weighted sum of DNAm beta values across the selected 91 CpGs in this sample—and BMI were then evaluated in STRATIFY using linear regression. No further STRATIFY association analyses were performed; this procedure served solely to confirm the transferability of the selected CpGs and weights.

## Results

### Identification of Distinct Longitudinal BMI Profiles Across Adolescence

The descriptive characteristics of the sample, which includes 1296 participants with available BMI data at age 14, are shown in Table 1. We applied longitudinal LPA to group participants based on their BMI from ages 14 to 23 (Methods and Materials; Supplement and Figure S2). This resulted in the identification of 2 distinct groups (Table S1): one, which consisted of participants overweight throughout adolescence (*n* = 200; 15.43% of the sample), and the other, of participants with persistently normal BMI (*n* = 1096) (Figure 1A). The 2 groups differed not only in BMI at each age but also in their overall changes, with the persistently overweight group showing a greater BMI increase across development (OR per 1 SD increase = 15.3, *p* < .001) (Methods and Materials) as well as increasing rates of obesity. There were no significant sex differences between the groups (χ^2^_1_ = 3.317, *p* = .069) (Table 1) and no differences in age at any time point (all *p* values > .05).

**Table 1:** Group Differences in BMI and ED-Related Behaviors and Concerns From Ages 14 to 23

Variable	Persistently Normal BMI Group, *n* = 1096	Persistently Overweight Group, *n* = 200	χ^2^_1_/OR (95% CI)	*p* Value	*p*_Bonferroni_
Sex, Female/Male	548 (50%)/548 (50%)	114 (57%)/86 (43%)	χ^2^_1_ = 3.04	.081	–

Age 14					
BMI	19.860 (2.247)	26.161 (3.205)	2.844 (2.463–3.348)	7.41 × 10^−41^	5.19 × 10^−40^∗
BMI Categories, % Within Each Group					
Underweight	2.8%	0.0%	–	–	–
Healthy weight	91.6%	18.6%			
Overweight	5.4%	55.3%			
Obesity	0.2%	26.1%			
Dieting	0.146 (0.353)	0.183 (0.388)	1.340 (0.787–2.230)	.269	>.999
Binge Eating	0.108 (0.311)	0.146 (0.354)	1.391 (0.763–2.464)	.268	>.999
Purging	0.085 (0.279)	0.088 (0.284)	1.012 (0.486–1.979)	.972	>.999
Eating Concerns	0.699 (1.182)	1.498 (1.620)	1.492 (1.333–1.671)	3.93 × 10^−12^	2.75 × 10^−11^∗
Weight and Shape Concerns	1.123 (1.620)	1.884 (1.753)	1.290 (1.175–1.415)	7.44 × 10^−8^	5.21 × 10^−11^∗
ED Concerns	1.822 (2.609)	3.381 (3.034)	1.215 (1.148–1.287)	2.69 × 10^−11^	1.89 × 10^−10^∗

Age 16					
Dieting	0.080 (0.272)	0.185 (0.389)	2.698 (1.620–4.422)	1.02 × 10^−4^	6.13 × 10^−4^∗
Binge Eating	0.078 (0.268)	0.115 (0.320)	1.293 (0.711–2.252)	.380	>.999
Purging	0.051 (0.221)	0.108 (0.312)	2.138 (1.131–3.895)	1.55 × 10^−2^	.093
Eating Concerns	0.818 (1.361)	1.389 (1.756)	1.243 (1.106–1.394)	2.22 × 10^−4^	1.33 × 10^−3^∗
Weight and Shape Concerns	1.140 (1.724)	1.815 (2.000)	1.198 (1.084–1.323)	3.93 × 10^−4^	2.36 × 10^−3^∗
ED Concerns	1.958 (2.887)	3.204 (3.451)	1.126 (1.061–1.194)	8.45 × 10^−5^	5.07 × 10^−4^∗

Age 19					
BMI	21.667 (2.451)	30.113 (4.455)	2.836 (2.370–3.516)	2.16 × 10^−25^	1.51 × 10^−24^∗
BMI Categories, % Within Each Group					
Underweight	5.9%	0.0%	–		
Healthy weight	90.0%	17.4%			
Overweight	4.0%	45.4%			
Obesity	0.1%	37.2%			
Dieting	0.071 (0.258)	0.151 (0.359)	2.300 (1.293–3.977)	3.52 × 10^−3^	.025∗
Binge Eating	0.065 (0.247)	0.079 (0.271)	1.031 (0.486–2.023)	.932	>.999
Purging	0.053 (0.223)	0.029 (0.168)	0.465 (0.136–1.207)	.159	>.999
Eating Concerns	0.757 (1.394)	1.425 (1.830)	1.285 (1.144–1.441)	2.00 × 10^−5^	1.40 × 10^−4^∗
Weight and Shape Concerns	0.996 (1.646)	1.612 (1.859)	1.199 (1.077–1.333)	8.23 × 10^−4^	5.76 × 10^−3^∗
ED Concerns	1.753 (2.868)	3.036 (3.282)	1.137 (1.069–1.209)	3.97 × 10^−5^	2.78 × 10^−4^∗

Age 23					
BMI	22.621 (2.795)	31.247 (5.000)	2.243 (1.950–2.643)	1.17 × 10^−25^	8.22 × 10^−25^∗
BMI Categories, % Within Each Group					
Underweight	3.0%	0.0%	–		
Healthy weight	92.0%	3.0%			
Overweight	5.0%	52.5%			
Obesity	0.0%	44.5%			
Dieting	0.032 (0.177)	0.093 (0.292)	2.999 (1.331–6.436)	5.82 × 10^−3^	3.49 × 10^−2^∗
Binge Eating	0.058 (0.233)	0.136 (0.344)	2.529 (1.299–4.743)	4.72 × 10^−3^	2.83 × 10^−2^∗
Purging	0.040 (0.196)	0.017 (0.130)	0.447 (0.070–1.585)	.286	>.999
Eating Concerns	0.602 (1.309)	1.220 (1.745)	1.288 (1.133–1.462)	9.13 × 10^−5^	5.48 × 10^−4^∗
Weight and Shape Concerns	0.738 (1.398)	1.271 (1.708)	1.229 (1.081–1.394)	1.46 × 10^−3^	8.76 × 10^−3^∗
ED Concerns	1.339 (2.528)	2.491 (3.210)	1.145 (1.068–1.228)	1.32 × 10^−4^	7.90 × 10^−4^∗

Logistic regression models were used to assess group differences, adjusting for sex and recruitment site. Bonferroni correction was applied for multiple testing across age, using a threshold of 0.05/7 phenotypes (or 0.05/6 for age 16, where BMI data were unavailable). BMI categories were derived using definitions from U.S. Centers for Disease Control and Prevention. For ages 14, 16, and 19, these were based on sex-specific BMI-for-age percentiles (https://www.cdc.gov/bmi/child-teen-calculator/bmi-categories.html). For age 23, categories are based on BMI range (https://www.cdc.gov/bmi/adult-calculator/bmi-categories.html).

BMI, body mass index; ED, eating disorder; OR, odds ratio.

∗ Bonferroni-adjusted *p* value < .05.

**Figure 1:**
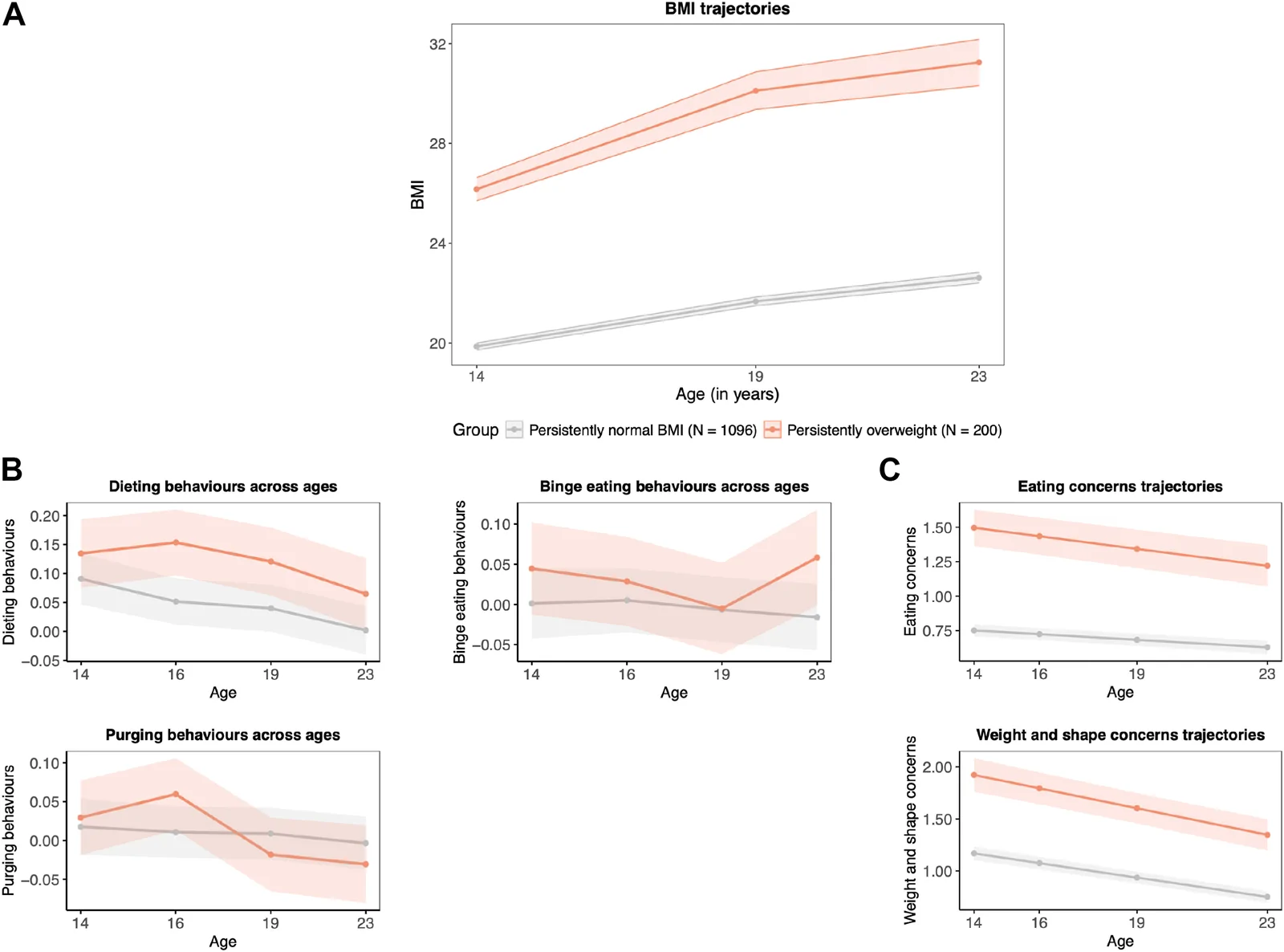
Identification of distinct BMI trajectory groups and their differences in eating disorder concerns and disordered eating behavior trajectories. **(A)** Two groups with distinct BMI trajectories across ages 14, 19, and 23 years were identified using latent profile analysis: a persistently overweight group (15.43% of the sample) and a persistently normal BMI group. In this panel, BMI at ages 14 and 19 cannot be directly compared with BMI at age 23, as the values were not adjusted for age- and sex-specific differences. **(B)** Trajectories of dieting, binge eating, and purging symptoms were analyzed using linear mixed models to assess group-by-age interactions. Data are presented as mean values with 95% CIs. **(C)** Group differences in eating concerns and weight and shape concerns from ages 14 to 23 were examined using conditional latent growth curve modeling. Predicted trajectories based on estimated intercepts and slopes are presented. Data are shown as mean values with 95% CIs; points along the lines represent mean values at each time point, and shaded areas represent 95% CIs. All models were adjusted for sex and recruitment sites. BMI, body mass index.

### Group Differences in ED-Related Behaviors and Concerns

Groups did not differ in DEBs at age 14. The persistently overweight group reported more dieting behaviors at age 16 (OR = 2.70), which persisted through age 23 (age 19: OR = 2.30; age 23: OR = 3.00), and more binge eating at age 23 (OR = 2.52; all *p*_Bonferroni_ < .001) (Figure 1B and Table 1). This behavioral pattern shows that the longitudinal BMI profiles capture the persistence of overweight across adolescence and reveal developmental changes in eating behavior that are not apparent when participants are classified solely according to BMI at age 14 (Supplement; Figures S3 and S4).

Unsurprisingly, significant group differences in eating and weight and shape concerns were observed at all ages (all *p*_Bonferroni_ < .001) (Figure 1C and Table S2). Compared with the persistently normal BMI group, the persistently overweight participants exhibited significantly higher levels of ED-related concerns at baseline (eating concerns: β = 0.652, *p* < .001; weight and shape concerns: β = 0.647, *p* < .001; total ED-related concerns: β = 1.301, *p* < .001). Despite these initial differences, both groups exhibited similar declines in ED-related concerns across adolescence and emerging adulthood, as indicated by nonsignificant slope estimates (all *p* values > .05).

### Group Differences in GMV, CT, and SD

Cross-sectional VBM analyses adjusted for TIV showed lower regional GMV in the persistently overweight group than in the normal BMI group (Figure S5 and Table S3). At age 14, lower volumes encompassed the left cerebellum, right paracentral lobule, and left caudate; by age 23, differences extended to bilateral insular regions (*p*_FWE_ < .001).

Longitudinal analyses identified 5 clusters showing divergent GMV trajectories between groups (*p*_FWE_ < .05) (Figure 2A and Table S4), including the middle temporal gyrus and orbitofrontal cortex (inferior frontal gyrus and gyrus rectus). Across these regions, the overweight group showed relative GMV increases, indicative of less pronounced reductions. Surface-based analyses (Figure 2B, C) further supported atypical neurodevelopmental trajectories, revealing thinner cortex and attenuated cortical thinning in occipital, parietal, and frontal regions, and initially higher but accelerated reductions in SD across sensorimotor and parietal cortices (all *p*_Bonferroni_ < .05) (Tables S5 and S6).

**Figure 2:**
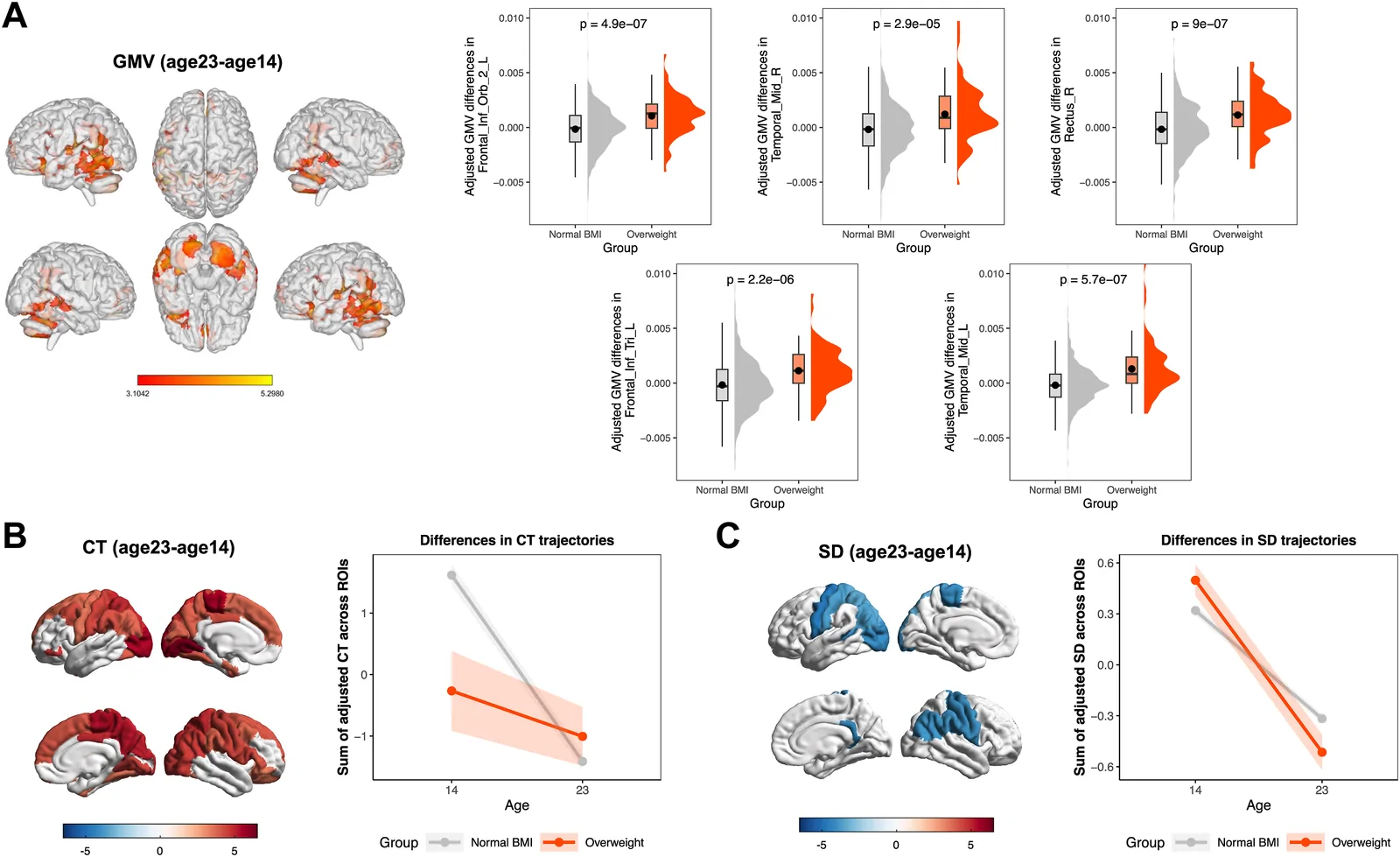
Group differences in GMV, CT, and SD maturation from age 14 to age 23. **(A)** Longitudinal analyses revealed significant group differences in GMV maturation between the persistently overweight and normal BMI groups. Regions showing relatively greater GMV increases in the overweight group included the left middle temporal gyrus (Temporal_Mid_L), right middle temporal gyrus (Temporal_Mid_R), left orbital part of the inferior frontal gyrus (Frontal_Inf_Orb_2_L), left triangular part of the inferior frontal gyrus (Frontal_Inf_Tri_L), and right rectus gyrus (Rectus_R). Box plots display the minimum and maximum values (whiskers), median (center line), and outliers (points beyond the whiskers) for each group. **(B)** Significant age-by-group interactions in longitudinal CT analyses showed less pronounced cortical thinning in the persistently overweight group, predominantly across occipital, parietal, and frontal regions. **(C)** Longitudinal analyses of SD revealed the opposite pattern, with more pronounced reductions in SD in the persistently overweight group, particularly in sensorimotor and parietal cortices. In panels **(B)** and **(C)**, solid lines represent mean values of the adjusted brain morphological measures (CT or SD), and shaded areas represent the corresponding 95% CIs. Across all panels, the y-axis indicates adjusted GMV, CT, or SD values after regressing out the effects of sex, recruitment sites, and total intracranial volume. CT, cortical thickness; GMV, gray matter volume; ROI, region of interest; SD, sulcal depth.

### Relationships Between DNAm and BMI

At age 14, 29 CpGs were epigenome-wide significant for BMI (*p*_Bonferroni_ < 1.3 × 10^−7^) (Figure 3A and Table S7), and 25,476 CpGs were nominally significant (Table S8). Most top associations were robust to smoking and negative life-event adjustments (Table S7). Six significant CpGs replicated a recent BMI EWAS (41). These were in regulatory elements, such as enhancers between the *PIM-1* proto-oncogene and immune cell–expressed *FGD2* gene (cg03957124); in *SBNO2*, a gene involved in the downstream anti-inflammatory effects of interleukin 10 (cg07573872) (42); in *VGLL4*, which encodes a positive regulator of the survival of human embryonic stem cells (cg18062721); and in the promoters of the *SKI* proto-oncogene (cg09469355), *ITGAL*, which encodes a leukocyte adhesion receptor (cg16391678), and *CALHM1*, which encodes a voltage-gated ion channel required for taste perception (cg26712428) (43). Among nominal associations, 510 previously significant CpGs showed >99.8% directional concordance (41).

**Figure 3:**
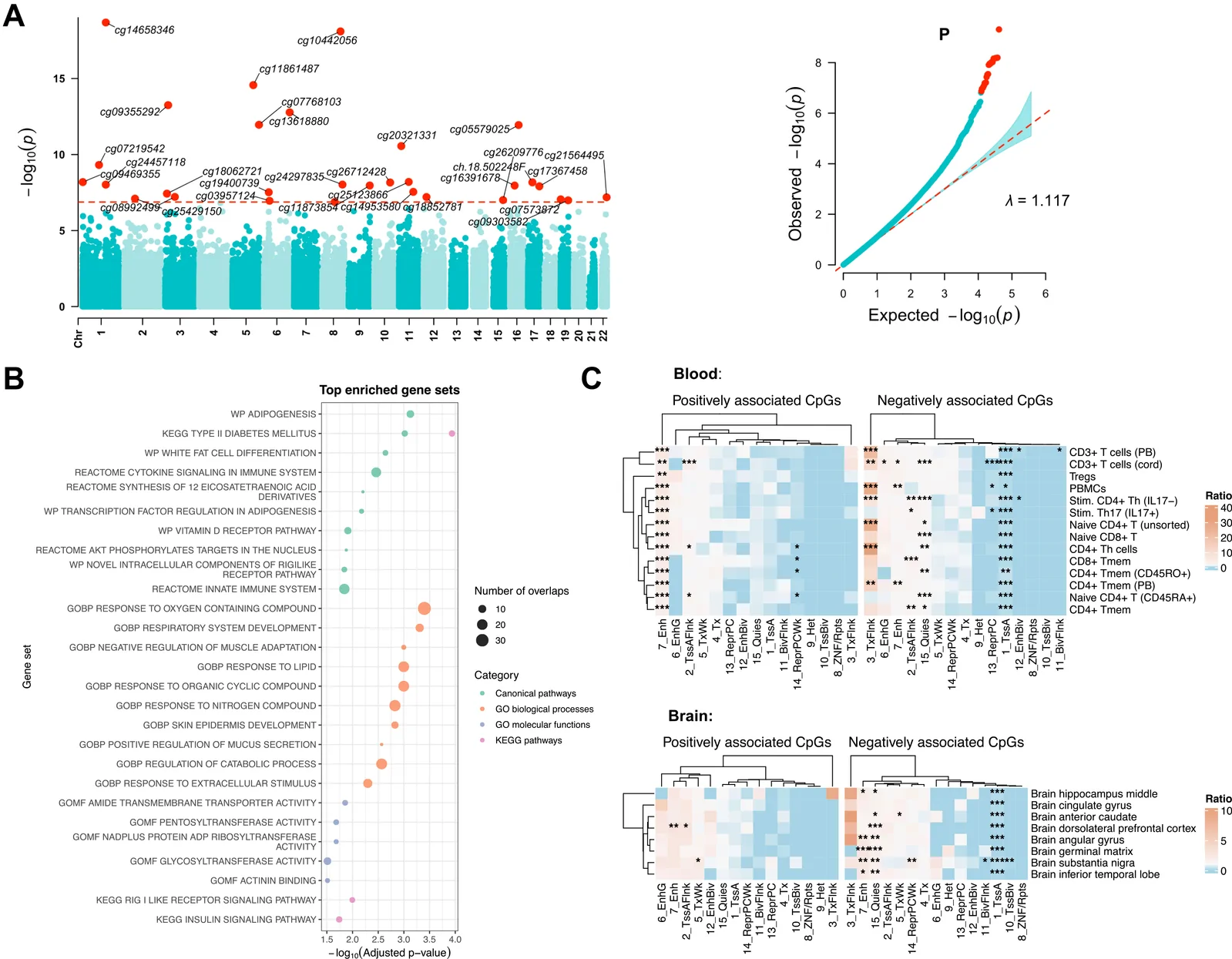
EWAS and enrichment analysis of CpGs associated with BMI. **(A)** Manhattan and QQ plots display the results of the BMI EWAS at age 14 years. The red dashed line in the Manhattan plots indicates the Bonferroni-corrected epigenome-wide significance threshold (*p* < 1.3 × 10^-7^). The shaded area in the QQ plots represents the 95% CI. **(B)** FUMA enrichment results are shown for FDR-significant CpGs negatively associated with BMI (*n* = 103). No significant enrichment findings were observed for the positively associated CpGs (*n* = 54). **(C)** Chromatin state enrichment analysis of all FDR-significant CpGs was performed separately for the positively and negatively associated CpGs across brain and blood tissues, based on the core 15-state ChromHMM model defined by the NIH Roadmap Epigenomics Mapping Consortium (https://egg2.wustl.edu/roadmap/web_portal/chr_state_learning.html#core_15state). Color indicates the enrichment ratio, with blue indicating lower and red indicating higher enrichment relative to background. CD3^+^ T cells (cord) indicates primary CD3^+^ T cells from cord blood; CD3^+^ T cells (PB), primary CD3^+^ T cells from peripheral blood; CD4^+^ Tmem (PB), primary CD4^+^ effector/memory T cells from peripheral blood (CD25int, CD127^+^); CD4^+^ Th cells, primary CD4^+^ T helper cells from peripheral blood (CD25–); CD4^+^ Tmem, primary CD4^+^ memory T helper cells (general population); CD4^+^ Tmem (CD45RO+), primary CD4^+^ memory T helper cells (CD45RO+) from peripheral blood; CD8^+^ Tmem, primary CD8^+^ memory T cells from peripheral blood; Naïve CD4^+^ T (CD45RA+), primary naïve CD4^+^ T helper cells (CD45RA+) from peripheral blood; Naïve CD4^+^ T (unsorted), primary naïve CD4^+^ T cells (unsorted) from peripheral blood; Naïve CD8^+^ (T), primary naïve CD8^+^ T cells from peripheral blood; Stim. CD4^+^ Th (IL17−), PMA-Ionomycin-stimulated CD4^+^ T helper cells (IL17−); Stim. Th17 (IL17+), PMA-Ionomycin-stimulated Th17 cells (IL17+); Tregs, primary CD4^+^ regulatory T cells from peripheral blood (CD25^+^, CD127–); and ZNF/Rpts, ZNF genes and repeats. ∗*p*_FDR_ < .05, ∗∗*p*_FDR_ < .01, ∗∗∗*p*_FDR_ < .001. ADP, adenosine diphosphate; BivFlnk, flanking bivalent TSS/enhancer; BMI, body mass index; chr, chromosome; Enh, enhancer; EnhG, genic enhancer; EnhBiv, bivalent enhancer; EWAS, epigenome-wide association study; FDR, false discovery rate; GOBP, gene ontology biological processes; GOMF, gene ontology molecular function; Het, heterochromatin; KEGG, Kyoto Encyclopedia of Genes and Genomes; PBMC, peripheral blood mononuclear primary cell; Quies, quiescent/low activity; ReprPC, repressed Polycomb; ReprPCWk, weak repressed Polycomb; TssA, active TSS; TssAFlnk, flanking active TSS; TssBiv, bivalent/poised TSS; Tx, strong transcription; TxFlnk, transcription at 5′ and 3′ gene ends; TxWk, weak transcription.

Analyses of DMRs identified 38 DMRs associated with BMI (Table S9). The most significant were located within *RAB26*, encoding a protein linking synaptic vesicles to the autophagy pathway (44), and genes involved in immune response [e.g., *ITGAL*, *TRAF7*, *TNF*, *TRIM31* (45), *IL32*, *PTPRCAP*, *CCR6*, and *SPI1*] or taste perception (*CALHM1*).

Functional enrichment analyses were performed using genes annotated to all FDR-significant CpGs associated with BMI (Table S10). No significant terms were identified for genes annotated to positively associated CpGs. Genes annotated to negatively associated CpGs were enriched for pathways related to adipogenesis, glucose metabolism, immune and cytokine signaling, inflammatory responses, and insulin signaling (all *p*_FDR_ < .05) (Figure 3B). Significant terms included adipogenesis, type 2 diabetes mellitus, white fat cell differentiation, cytokine signaling, innate immune system pathways, interleukin signaling, RIG-I–like receptor signaling, and insulin signaling. Gene ontology terms also highlighted lipid and nutrient responses, carbohydrate metabolism, catabolic processes, immune regulation, apoptosis, and fat cell differentiation. Overall, these results suggest that negatively associated BMI CpGs map to genes involved in metabolic and immune-related processes.

Tissue-specific expression analyses revealed enrichment for genes expressed at lower levels in brain tissues, with the highest enrichment in the hypothalamus, hippocampus, basal ganglia, and amygdala (Figure S6). Chromatin state analyses (Figure 3C and Table S11) showed that CpGs positively associated with BMI were enriched in enhancer regions across blood T-cell populations, with additional enrichment in regions flanking active transcription start sites. In brain tissues, positively associated CpGs were enriched in enhancers, flanking active transcription start sites, and weakly transcribed regions. CpGs negatively associated with BMI were consistently depleted from active transcription start sites across brain and blood tissues and cell types. In contrast, these CpGs were enriched in regions flanking actively transcribed genes, enhancer regions, and quiescent chromatin states. Additional enrichment was observed in weakly transcribed regions and weak PolyComb-repressed regions in specific brain tissues. Overall, BMI-associated CpGs were enriched in distal regulatory elements and, for negatively associated CpGs, in quiescent chromatin states, while being depleted at active promoters across multiple tissues.

To summarize the combined contribution of BMI-associated methylation variation, we constructed a bmiMRS using LASSO-penalized regression with 10-fold cross-validation (Methods and Materials and Table S12). In the IMAGEN cohort, the cross-validated bmiMRS explained 2.22% of the variance in BMI residuals (out-of-sample *R*^2^ = 0.0222, *p* = 9.50 × 10^−8^), with a correlation of *r* = 0.15 between observed and predicted values (MSE = 11.34). Application of the same CpGs and weights to the independent STRATIFY cohort showed a significant association with BMI, explaining 1.44% of the variance (*p* = .019) (Figure S7). Within IMAGEN, higher bmiMRS values were associated with higher BMI at age 14 (β = 0.16, *p* = 1.12 × 10^−8^), increased odds of belonging to the persistently overweight group (OR = 1.54, *p* = 1.60 × 10^−3^), and greater BMI AUC across adolescence (β = 0.16, *p* = 1.59 × 10^−4^), indicating that adolescents with higher methylation risk scores exhibited consistently higher BMI across adolescence and emerging adulthood.

### Associations Between Cortical Maturation, BMI, and DNAm

To probe the dynamic links between metabolic and neural development, we examined bidirectional longitudinal associations between BMI and GMV, CT, and SD in regions differentiating the BMI profile groups (Table S13).

Higher BMI at age 14 predicted greater GMV increases across all 5 regions differentiating the BMI profile groups, including the bilateral middle temporal and inferior frontal cortices (*r* > 0.20, all *p*_Bonferroni_ < 1 × 10^−7^), even after adjustment for baseline brain structure, indicating that early adiposity predicts later volumetric maturation independently of preexisting neuroanatomical differences. Such widespread associations were not observed for CT or SD, suggesting that early metabolic status selectively influences volumetric expansion or delayed pruning during adolescence. Associations were also observed between the bmiMRS and GMV change, but not CT or SD change. Without adjustment, the bmiMRS was positively associated with GMV increases in the right middle temporal gyrus (*r* = 0.115, *p*_Bonferroni_ = .032). However, after adjustment for baseline GMV, this association reversed direction (*r* = −0.102) and no longer survived Bonferroni correction (*p*_Bonferroni_ = .096). A similar pattern was observed for the left middle temporal gyrus, where the association became negative and significant (*r* = −0.113, *p*_Bonferroni_ = .049). This pattern suggests that higher methylation risk reflects baseline anatomical differences rather than a robust effect of methylation on subsequent volumetric change.

Conversely, baseline CT, but not GMV, predicted subsequent BMI increases independently of baseline BMI, with a thinner cortex across widespread occipital, parietal, sensorimotor, and frontal regions associated with greater weight gain during adolescence. Baseline SD showed only localized associations in postcentral and superior parietal regions, where greater SD predicted steeper BMI increases.

## Discussion

This study provides a multimodal perspective on adolescent BMI development by integrating longitudinal BMI profiles with behavioral, neuroimaging, and epigenetic data. Adolescents with persistent overweight showed progressively greater BMI, increased disordered eating behaviors, widespread differences in cortical maturation, and distinct DNAm signatures enriched for metabolic and inflammatory pathways. Importantly, longitudinal analyses suggested reciprocal associations between adiposity and brain development: Higher BMI in early adolescence predicted subsequent regional GMV maturation, whereas a thinner cortex at baseline independently predicted greater BMI increases during follow-up. Together, these findings support a developmental model in which behavioral, neurodevelopmental, and peripheral molecular processes jointly contribute to persistent overweight across adolescence.

Adolescents with persistent overweight reported higher eating-, weight-, and shape concerns, together with more frequent dieting and binge eating during follow-up. These findings are consistent with longitudinal evidence that weight stigma, body dissatisfaction, and restrictive eating behaviors increase vulnerability to binge eating and subsequent weight gain (8,46, 47, 48, 49, 50, 51). The absence of group differences in dieting behavior at age 14, followed by their emergence during adolescence, further suggests that maladaptive eating behaviors may develop progressively alongside persistent overweight rather than simply reflecting baseline weight status. These observations emphasize the importance of addressing both psychological distress and eating behaviors in early obesity prevention.

Our longitudinal BMI profiles are consistent with previous developmental studies that identified stable normal-weight and persistently overweight groups associated with increased cardiometabolic risk (52). Likewise, BMI-associated CpGs were enriched for genes involved in adipogenesis, type 2 diabetes, tumor necrosis factor (TNF) signaling, and immune regulation, pathways central to obesity-related metabolic inflammation (53). TNF has emerged as a key adipokine linking immune activation with energy homeostasis (54,55), and the chronic activation of TNF-dependent pathways contributes to the low-grade inflammatory state underlying insulin resistance and type 2 diabetes (56,57). Chromatin-state enrichment further indicates that BMI-associated CpGs preferentially localize to enhancer and other distal regulatory regions, consistent with epigenetic modulation of gene regulation rather than promoter methylation. Together with the enrichment of genes differentially expressed in brain tissues, these findings support growing evidence that obesity-related peripheral inflammation may influence central nervous system function through mechanisms including blood-brain barrier dysfunction and neuroinflammation (58).

The 91-CpG bmiMRS was designed for prediction rather than causal locus identification. Its highest-weighted CpGs mapped to genes involved in lipid metabolism, cellular stress, energy use, and hormone signaling — central to obesity (59). Negatively weighted CpGs highlighted loci involved in energy balance and insulin sensitivity, including *PKLR*, a rate-limiting enzyme in glycolysis and hepatic glucose metabolism (60), *PYGM*, a key enzyme in glycogen breakdown, and *PIK3IP1*, a negative regulator of the PI3K signaling pathway, which regulates metabolic homeostasis and insulin sensitivity (60, 61, 62). Positively weighted CpGs mapped to genes involved in vitamin D metabolism (*CYP2R1*) (63), stress-inducible regulation of mTOR (mechanistic target of rapamycin) signaling and insulin sensitivity (*SESN3*) (64,65), oxidative stress (*GSTM2*), and calcium signaling and taste perception (*CALHM1*) (43). Notably, *CALHM1*, together with loci annotated to *SBNO2*, *VGLL4*, and *SKI*, replicated a large BMI EWAS (41). Although its explained variance was modest, external association in STRATIFY supports some transferability, while the convergent pathways support biological relevance.

Neuroimaging analyses provided complementary evidence of altered cortical maturation in adolescents with persistent overweight. Relative GMV increases within the bilateral middle temporal gyri, left inferior frontal gyrus, and right gyrus rectus suggest delayed maturation of association cortices that normally undergo substantial gray matter reduction during adolescence. These regions support reward valuation (66), sensory-emotional integration (67,68), and inhibitory control (69), functions previously implicated in obesity and food cue responsivity (70,71).

Surface-based analyses further demonstrated attenuated cortical thinning across posterior and frontal regions, together with accelerated reductions in SD within the sensorimotor and parietal cortices. Because cortical thinning during adolescence is thought to reflect synaptic pruning and progressive myelination (72,73), slower thinning may indicate delayed maturation of executive and visuospatial networks, whereas altered sulcal morphology may reflect atypical cortical organization underlying body representation and visuospatial integration (74,75).

Importantly, several neuroanatomical differences were already present at age 14, indicating that divergence in brain development preceded the imaging follow-up period. Although earlier neuroimaging data were unavailable, the longitudinal analyses provide important insight into the temporal relationship between adiposity and brain maturation. Higher BMI at age 14 predicted subsequent regional GMV changes, whereas a thinner cortex at baseline independently predicted greater BMI increases across adolescence, even after adjustment for baseline BMI. In contrast, associations between the bmiMRS and longitudinal GMV change were attenuated after accounting for baseline GMV, suggesting that the observed methylation-brain relationships were largely explained by preexisting neuroanatomical differences rather than subsequent brain maturation. Collectively, these findings are most consistent with a reciprocal developmental model in which early adiposity influences subsequent neurodevelopment, while individual differences in cortical maturation independently contribute to later weight gain. Future studies incorporating repeated neuroimaging, DNAm, and metabolic assessments from childhood onward will be required to disentangle these dynamic relationships and establish causal mechanisms.

While the longitudinal design and integration of behavioral, neuroimaging, and epigenetic data represent major strengths, several limitations should be acknowledged. First, the cohort was restricted to individuals of European ancestry, limiting generalizability to other populations. Second, DNAm was measured only once, in whole blood at age 14, and should therefore be interpreted as identifying peripheral biomarkers associated with later outcomes rather than causal biological mechanisms. Third, enrichment analyses relied on annotated gene sets and external chromatin-state reference maps that may not fully account for probe density, genomic clustering, or developmental differences between reference tissues and the adolescent brain. Fourth, although all available longitudinal data were used, selective attrition may have introduced bias. Finally, dietary intake was unavailable, and therefore its contribution to both BMI development and DNAm could not be evaluated.

### Conclusions

Persistent adolescent overweight was accompanied by behavioral, neurodevelopmental, and epigenetic alterations. These peripheral biomarkers and brain-developmental signatures may inform earlier risk stratification and biologically grounded, developmentally tailored obesity prevention strategies.

## Supplementary Material

Tables S1–S13

Key Resources Table
