# Supplementary material for: Persistent Body Mass Index Profiles During Adolescence: Associations With Brain Maturation and DNA Methylation
Source: Biol Psychiatry Glob Open Sci. 2026 Sep 1;6(6):100820. doi: 10.1016/j.bpsgos.2026.100820 (PMC13693072; doi:10.1016/j.bpsgos.2026.100820)
Supplement: Supplementary file 1 [file mmc1.pdf]

SUPPLEMENTARY INFORMATION

Persistent Body Mass Index Profiles During Adolescence: Associations With Brain Maturation and DNA Methylation

Yu *et al.*

Supplementary Information

Table of Contents

SUPPLEMENTARY METHODS **3**

Behavioural questionnaires 3
Eating disorder concerns questionnaire: item details *3*
Disordered eating behaviours (DEBs) measure: item details *3*
Smoking, and negative life events (NLEs) measures *4*
BMI measurements 4
Longitudinal latent profile analysis (LPA) with BMI 5
Neuroimaging data processing and quality control 6
Structural Magnetic Resonance Imaging (MRI) acquisition *6*
Grey matter volumes (GMV) *7*
Surface-based measures: cortical thickness (CT) and sulcal depth (SD) *8*
DNA methylation processing 9
DNA extraction and microarray hybridisation *9*
DNA methylation preprocessing and quality control *9*
Quality assessment and control for confounders *10*
Relatedness and ethnicity 10

SUPPLEMENTARY FIGURES **11**

Figure S1. Analytical flowchart of the study. 11
Figure S2. Comparison of latent BMI profile solutions. 13
Figure S3. Different longitudinal BMI profile solutions and associated disordered eating behaviours. 14
Figure S5. Cross-sectional GMV differences between the persistently high and normal BMI groups. 17
Figure S6. Tissue-specific enrichment of all FDR-significant CpGs associated with BMI. 19
Figure S7. Cross-validation and external validation of the methylation risk score for BMI (bmiMRS). 20
Figure S8. Principal component analysis (PCA) of DNA methylation levels across all autosomal probes. 21
Figure S9. Effects of array location on DNA methylation. 22
Figure S10. Effects of different processing waves on DNA methylation. 23
Figure S11. Estimated blood cell-type proportion. 24

SUPPLEMENTARY REFERENCES **25**

Supplementary methods

Behavioural questionnaires

Eating disorder concerns questionnaire: item details

The following questions were used to assess eating concerns, and weight and shape concerns, according to section P of the Development and Well-Being Assessment (DAWBA questionnaire) (1).

For eating concerns,

- Would you be ashamed if other people know how much you eat?
- Do worries about eating (what? where? how much?) really interfere with your life?
- If you eat too much, do you blame yourself a lot?
- Do you spend a lot of your time thinking about food?
- Sometimes people say that they have such a strong desire for food, and that this desire is so hard to resist, that it is like the way an addict feels about drugs or alcohol. Does this apply to you?

For weight and shape concerns,

- Have you ever thought you were fat even when other people told you that you were very thin?
- Are you afraid of gaining weight or getting fat?
- Does the thought of gaining weight or getting fat really terrify you?
- If a doctor told you that you needed to put on five pounds (two kilograms) for the sake of your health, would you find this easy, difficult or impossible to accept?

To address concerns related to eating disorders (ED), we computed the total scores of eating concerns and weight and shape concerns.

Disordered eating behaviours (DEBs) measure: item details

Binary variables for DEBs, including dieting, binge eating, and purging behaviours were derived from the following questions in section P of the DAWBA questionnaire.

For dieting behaviours,

- If the participant responded “a lot” to any of the following statements recording the frequency of their eating behaviour: (1) eating less at meals; (2) skipping meals; (3) going without food for long periods, e.g., all or most of the day.

For binge eating behaviours,

- If the participant responded “yes” to the question: sometimes people lose control over what they eat, and then they eat a very large amount of food in a short time. For example, they may open the fridge and eat as much as they can find – eating and eating until they feel physically ill. This usually happens when people are by themselves. Does this happen to you?

For purging behaviours,

- If the participant responded “yes” to any of the following statements: (1) have you ever deliberately made yourself vomit (throw up)? (2) over the past three months, have you done any of the following to avoid putting on weight: vomit/taking pills or medicine in order to lose weight?

Smoking, and negative life events (NLEs) measures

The frequency of cigarette consumption over a lifetime was evaluated by the response to the following question: “On how many occasions during your lifetime have you smoked cigarettes?”, as part of the Fagerstrom Test for Nicotine Dependence (2).

NLEs were assessed using the life events questionnaire, an adaptation of the stressful life-event questionnaire (3). Twenty events, classified into seven subscales, including family/parents, accident/illness, sexuality, autonomy, deviance, relocation, and distress, were defined as negative or stressful based on self-reports from participants who had experienced the event and rated it as distressing (4). The total lifetime frequency of NLEs was used in the analysis.

BMI measurements

The BMI categories were identified using definitions from the U.S. Centres for Disease Control and Prevention (CDC). At ages 14 to 19, sex- and age-standardised BMI z-scores were generated to derive BMI percentiles based on sex-specific BMI growth charts (https://www.cdc.gov/bmi/child-teen-calculator/bmi-categories.html). Categories were

defined as follows: Underweight: below the 5th percentile; Healthy weight: 5th to <85th percentile; Overweight: 85th to <95th percentile; Obesity: ≥95th percentile. At age 23, categories were based on BMI ranges (https://www.cdc.gov/bmi/adult-calculator/bmi-categories.html): Underweight: <18.5; Healthy weight: 18.5 to <25; Overweight: 25 to <30; Obesity: ≥30. To capture changes in BMI from ages 14 to 23, we calculated the area under the curve (AUC) for BMI using the trapezoidal method, which integrates BMI values across the three assessments (i.e., ages 14, 19 and 23) weighted by the time interval between assessments.

Longitudinal latent profile analysis (LPA) with BMI

Longitudinal LPA was used to identify latent subgroups based on BMI at ages 14, 19, and 23 years, with sex and recruitment site included as covariates (**Table S1**). Models with two, three, and four classes were evaluated using the Bayesian Information Criterion (BIC), Akaike Information Criterion (AIC), entropy, and the Lo-Mendell-Rubin likelihood ratio test (LMR-LRT), which compares models with k versus k−1 classes. Selection of the optimal solution was based on a combination of statistical fit, class separation, class size, profile distinctiveness, model stability, and interpretability. Lower AIC and BIC values indicate better model fit, while entropy values greater than 0.80 indicate good class separation (5). Solutions containing classes representing less than 5% of the sample were considered potentially unstable (6). Preference was also given to solutions in which classes reflected distinct developmental patterns rather than subdivisions of an existing profile.

Visualisations of the BMI and disordered eating behaviours profiles for the 2-, 3-, and 4-class solutions (**Figs. S2 and S3**) indicated that although the 3- and 4-class models showed modest improvements in fit indices, these solutions primarily subdivided the elevated BMI profile into groups differing in BMI level rather than identifying qualitatively distinct patterns of BMI change over time, indicating that the principal source of heterogeneity in the IMAGEN cohort was BMI level rather than profile shape. In addition, although the 3- and 4-class models identified additional BMI subgroups, the behavioural profiles of these classes were broadly similar. The most consistent behavioural differences were observed between participants with persistently elevated BMI and those with normal BMI, whereas the additional classes largely represented subdivisions of the elevated BMI group with overlapping behavioural characteristics. Therefore, considering statistical fit, class stability, interpretability, and

parsimony, the two-class solution was retained, comprising a persistently normal BMI group and a persistently overweight BMI group.

We performed additional analyses using a conventional binary baseline weight-status classification instead of the longitudinal BMI-profile classification. Participants were classified according to CDC BMI-for-age criteria at age 14 as normal weight (5th to <85th percentile) or overweight/obese (≥85th percentile).

Overall, both this baseline BMI classification and the LPA approach identified adolescents at greater risk for disordered eating. However, they did not produce identical findings. The most informative difference concerned dieting behaviour. Under the baseline BMI classification, adolescents with overweight/obesity already reported significantly greater dieting behaviour at age 14, reflecting contemporaneous differences associated with current weight status. In contrast, the longitudinal BMI-profile analysis showed no group differences in dieting behaviour at age 14. Instead, differences first emerged at age 16 and persisted into early adulthood, suggesting that they capture different aspects of BMI development.

Neuroimaging data processing and quality control

Structural Magnetic Resonance Imaging (MRI) acquisition

High-resolution structural MRI images were acquired from eight IMAGEN recruitment sites using 3 Tesla MRI scanners from different manufacturers (Siemens, Munich, Germany; Philips, Best, The Netherlands; General Electrics, Chalfont St Giles, UK; Bruker, Ettlingen, Germany). Scanning parameters were selected to ensure compatibility across all scanners, and imaging protocols were harmonised across sites. T1-weighted three-dimensional anatomical images were acquired using a magnetisation-prepared rapid acquisition gradient echo (MPRAGE) sequence, following the ADNI protocol (https://adni.loni.usc.edu/data-samples/adni data/neuroimaging/mri/mri-scanner-protocols/), with an isotropic voxel size of 1.1 mm. T2-weighted fast-spin echo and FLAIR scans were also collected to allow any incidental findings to be reviewed.

Grey matter volumes (GMV)

Cross-sectional preprocessing: T1-weighted images were preprocessed using the Statistical Parametric Mapping toolbox (SPM12; Wellcome Centre for Human Neuroimaging, UCL, London, UK), implemented in MATLAB (v.2022b; MathWorks, Natick, MA, USA). After manual reorientation to align the anterior commissure with the origin of the Montreal Neurological Institute (MNI) coordinate system, images were segmented into grey matter, white matter, and cerebrospinal fluid using the VBM8 toolbox (http://www.neuro.uni-jena.de/vbm), which applies the unified segmentation algorithm incorporating prior tissue probability maps. Segmented grey matter maps were then used to construct a study-specific template using the DARTEL algorithm (7). Spatial normalisation to MNI space (1.5 × 1.5 × 1.5 mm resolution) was performed via iterative diffeomorphic registration, and modulated grey matter maps were smoothed using an 8 mm full-width at half maximum (FWHM) Gaussian kernel. Final voxel values represented the proportion of grey matter volume at each location, enabling voxel-based morphometry (VBM) analyses at ages 14 and 23.

Longitudinal pre-processing: Longitudinal structural images (ages 14 and 23) were processed using the pairwise registration pipeline in SPM12 (8), which minimises bias from asymmetric warping and intransitive transformations. For each participant, a within-subject average template was generated through combined correction of intensity inhomogeneity, rigid and diffeomorphic registration, and template generation. This produced Jacobian Determinant (JD) maps quantifying the annualised voxel-wise rate of volumetric change across the nine-year interval. Grey matter maps were multiplied voxel-wise by JD maps, normalised to MNI space using DARTEL flow fields, and smoothed with an 8 mm FWHM Gaussian kernel. Final voxel values reflected the local rate of grey matter volume change per year.

Quality control (QC) and total intracranial volume (TIV): Image quality was assessed with the MRIQC toolbox (9), which provides a comprehensive set of quality metrics, including measures based on noise measurements (coefficient of joint variation and contrast-to-noise ratio), information theory (entropy of voxel intensities and the mean energy of image values within the head relative to outside the head), measures targeting specific artefacts (summary statistics of the bias field, the proportion of voxels with intensity corrupted by artefacts normalised by the number of voxels in the background), and other measures (the FWHM of the spatial distribution of the image intensity values in units of voxels, and the mean, median, median absolute deviation, standard deviation, kurtosis, 5% percentile, 95% percentile and a

number of voxels of the distribution of background, cerebrospinal fluid, grey matter and white matter). JD maps with grey matter values deviating more than three standard deviations from the group mean were flagged for manual review, and scans showing artefacts or abnormal morphology were excluded. Total intracranial volume (TIV) was estimated for each participant at both time points using VBM8 segmentations. For cross-sectional analyses, TIV at the corresponding age was included as a covariate; for longitudinal analyses, the difference between ages 14 and 23 (ΔTIV) was included to account for global brain growth.

Surface-based measures: cortical thickness (CT) and sulcal depth (SD)

In addition to GMV, we derived surface-based measures of CT and SD –a measure of cortical folding or gyrification. These distinct neuroanatomical measures provide complementary information on cortical architecture, allowing a more comprehensive assessment of structural brain development.

Longitudinal preprocessing and QC: Raw images were visually inspected to exclude images with movement artefacts, brace artefacts or field inhomogeneities before preprocessing. Preprocessing was conducted using the Computational Anatomy Toolbox (CAT 12.8 (r1907); https://neuro-jena.github.io/cat/) in SPM 12 (Wellcome Department of Cognitive Neurology). The 'segment longitudinal data' pipeline was applied with default settings. Intrasubject coregistration was performed on baseline (age 14) and follow-up (age 23) images, which were then bias-corrected relative to the mean image. Baseline, follow-up, and mean images were segmented into grey matter, white matter, and cerebrospinal fluid using default tissue priors. Segmented mean images were normalised to MNI space using DARTEL, and the derived parameters were applied to baseline and follow-up grey matter images. Normalised images were smoothed with a 6 mm FWHM Gaussian kernel. Participants were excluded if MRI data were missing or if QC criteria were not met (images with quality below grade D were excluded).

Measures and regions of interest (ROIs): After preprocessing, surface-based measures of CT and square root-transformed SD were computed using CAT12 and resampled to 12 mm mesh resolution, as recommended for surface metrics. Longitudinal changes in mean CT and SD were extracted for 68 ROIs defined by the Desikan–Killiany atlas.

DNA methylation processing

DNA extraction and microarray hybridisation

Genomic DNA was extracted from whole blood samples (~10 mL), collected from participants at age 14 and preserved in BD Vacutainer EDTA tubes (Becton, Dickinson and Company), using the Gentra Puregene Blood Kit (QIAGEN) in accordance with the manufacturer's protocol. DNA methylation (DNAm) profiling was performed using the Infinium HumanMethylation450 BeadChip (450K array), which interrogates over 485,000 CpG sites across the genome. Sample processing and hybridisation were conducted in two separate waves. In the first wave, 708 samples were randomly selected from the full cohort; the remaining samples were processed in the second wave. To adjust for any batch effects introduced by this two-wave design, wave membership was included as a covariate in all subsequent statistical analyses.

DNA methylation preprocessing and quality control

Prior to analysis, samples with insufficient DNA quantity, duplicated entries, or mislabelling during extraction were excluded. Raw methylation intensities were processed using the minfi R package(10). Intensities were converted to β values, defined as the proportion of methylated signal intensity to the sum of methylated and unmethylated intensities at each locus ($\beta = M / (M + U)$. Initial quality assessment was conducted using the *preprocessIllumina* function. Outlier detection was based on principal component analysis (PCA) of autosomal probes, with samples more than three standard deviations from the median on the first four PCs excluded. Predicted sex, derived from sex chromosome probe intensities, was compared with reported sex, and discordant samples were removed. Data generated in separate waves were merged prior to further analysis.

Within- and between-sample normalisation was performed using a stratified quantile normalisation in minfi. Probes were excluded if they: (i) mapped to sex chromosomes, (ii) failed detection in >20% of samples (detection p-value > 0.01), (iii) contained a common SNP (minor allele frequency ≥ 0.05) at the CpG or single base extension site, or (iv) were annotated as cross-reactive (11). To reduce false discoveries and improve statistical power (12), non-variable probes –defined as probes with β values consistently ≤ 0.2 or ≥ 0.8 across participants– were also filtered out. After all filtering steps, 372,582 CpG probes remained for analysis.

Quality assessment and control for confounders

PCA of autosomal DNAm levels (**Figure S7**) showed that the first four PCs captured major sources of technical variation and were retained as covariates in downstream analyses. No significant effects of sample location on arrays were observed (**Figure S8**). Differences between processing waves were evident in these PCs (**Figure S9)**, and the wave membership was therefore included as an additional covariate. Cell composition was estimated for six major blood cells (granulocytes, B cells, CD4+ T cells, CD8+ T cells, monocytes, and natural killer cells). PCA of the estimated proportions revealed that the first two components explained 85% of the total variance (**Figure S10**) and were included as covariates to efficiently adjust for compositional effects while avoiding multicollinearity from directly modelling all cell types. Finally, to control for potential population stratification, the first four multidimensional scaling (MDS) components derived from genome-wide genotype data were also included as covariates.

Relatedness and ethnicity

Detailed information on participant relatedness and ethnicity is provided in (4,13). Genetic relatedness and population structure were further evaluated by computing pairwise kinship coefficients using the KING software (14). To account for familial relationships and accurately characterise population structure, principal component analysis (PCA) was conducted using the GENESIS R package (15), which models genetic structure while controlling for relatedness.

The resulting principal components were used to identify and visualise genetic ancestry clusters. Participants of non-European ancestry—identified as outliers in the PCA plot relative to the main European ancestry cluster of the IMAGEN cohort—were excluded from downstream analyses to minimise population stratification effects.

Supplementary figures

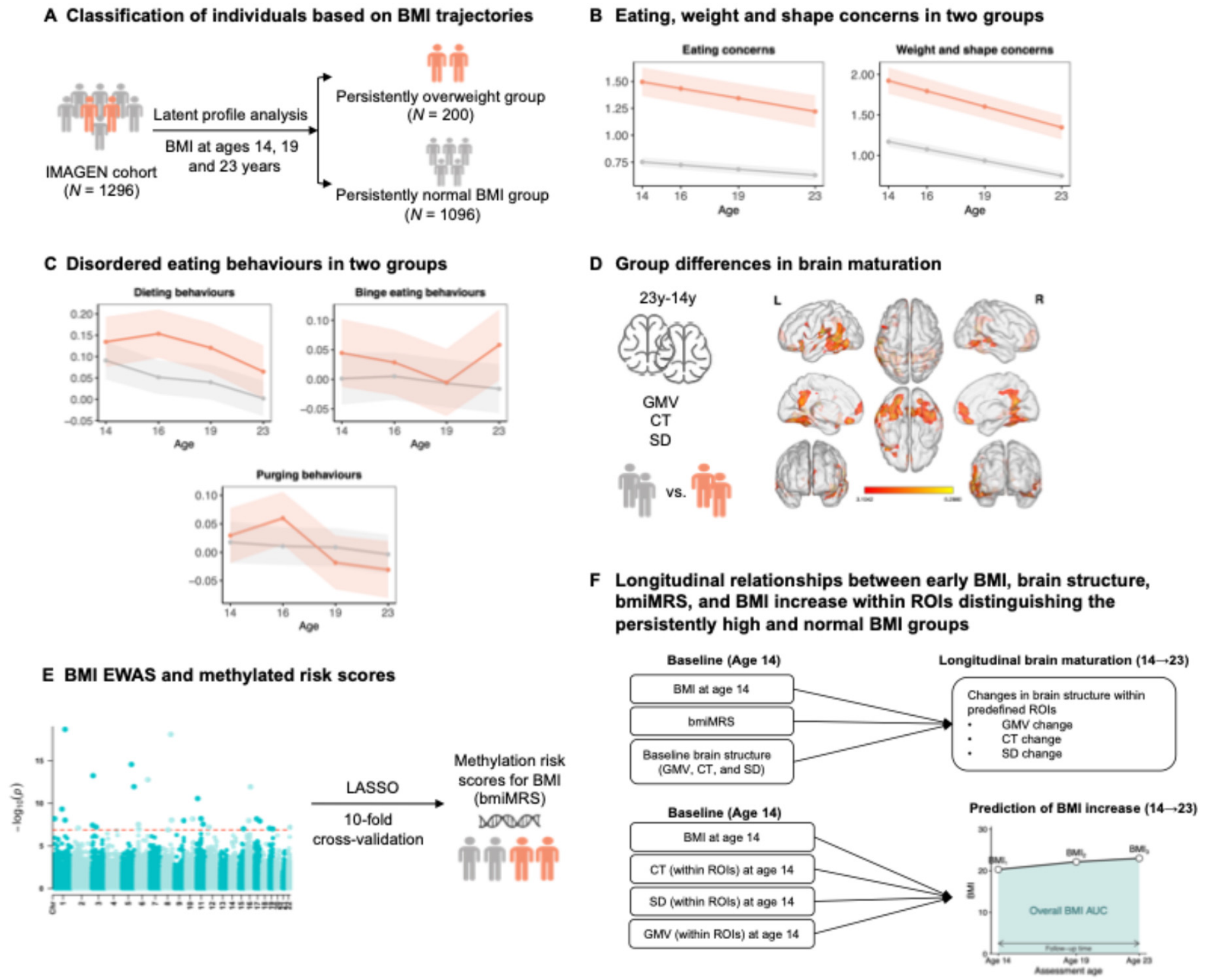

Figure S1. Analytical flowchart of the study.

Overview of the analytical pipeline used to investigate longitudinal BMI profiles and their behavioural, neurodevelopmental, and epigenetic correlates in the IMAGEN cohort (N = 1,296). **(A)** Latent profile analysis of BMI measured at ages 14, 19, and 23 years identified two stable BMI profiles: a persistently overweight group (N = 200) and a persistently normal BMI group (N = 1,096). **(B)** Longitudinal trajectories of eating concerns and weight/shape concerns were compared between the two BMI profile groups. **(C)** Group differences in disordered eating behaviours (dieting, binge eating, and purging) were examined across adolescence. **(D)** Longitudinal neuroimaging analyses assessed differences in brain maturation between groups using grey matter volume (GMV), cortical thickness (CT), and sulcal depth (SD). **(E)** Epigenome-wide association studies (EWAS) of BMI at age 14 identified BMI-associated CpG sites, which were subsequently used to derive a methylation risk score for BMI (bmiMRS) using LASSO regression with 10-fold cross-validation. **(F)** Longitudinal association analyses examined bidirectional relationships between BMI, BMI-

associated DNA methylation (bmiMRS), and brain structure within ROIs distinguishing the persistently high and normal BMI groups.

GMV, grey matter volume; CT, cortical thickness; SD, sulcal depth; EWAS, epigenome-wide association study; BMI, body mass index; PGS, polygenic scores. LASSO, least absolute shrinkage and selection operator. AUC, area under the curve.

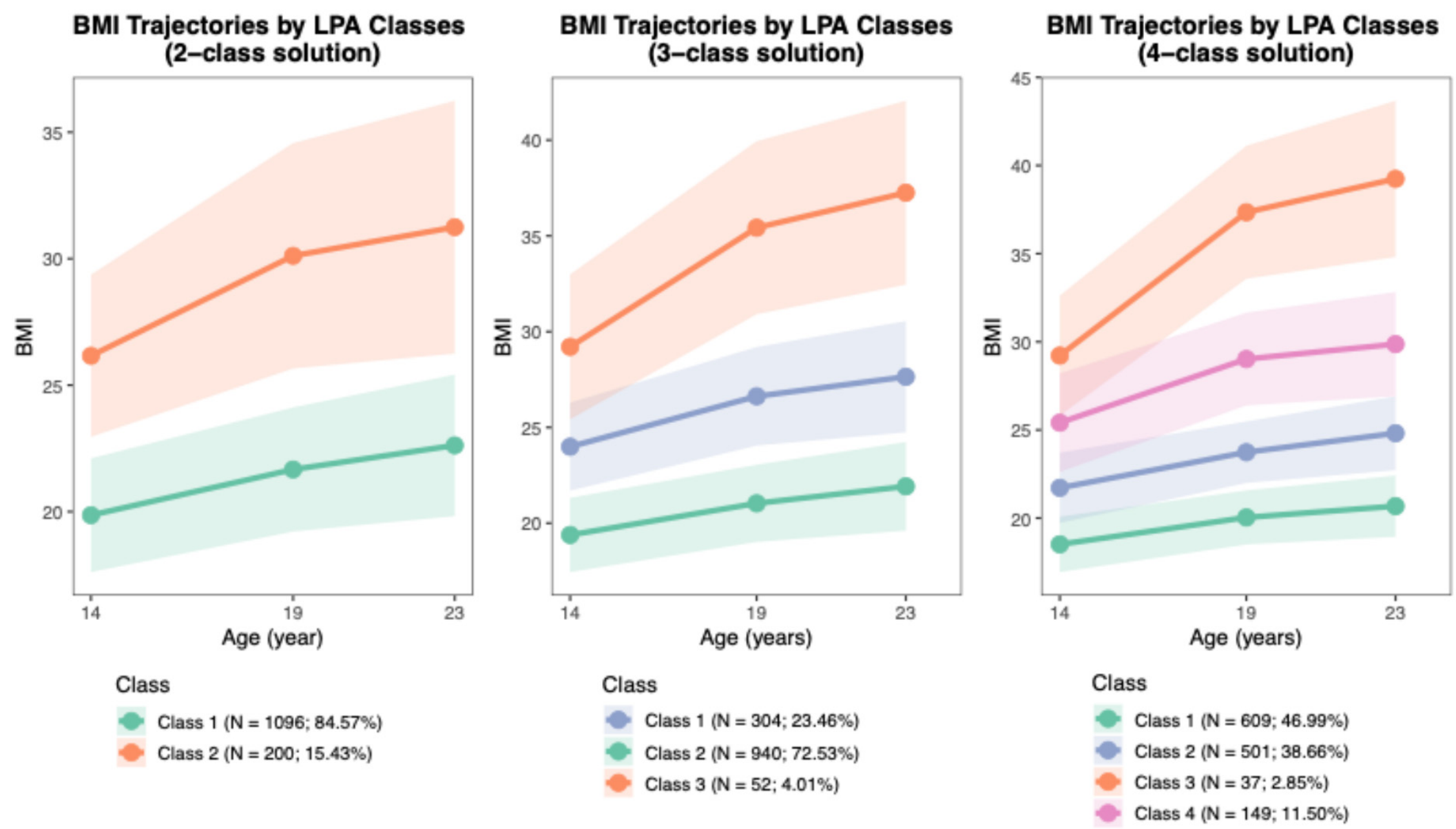

Figure S2. Comparison of latent BMI profile solutions.

Longitudinal BMI profiles identified using 2-, 3-, and 4- class latent profile analysis (LPA) models based on BMI measured at ages 14, 19, and 23 years. Lines represent mean BMI profiles for each latent class, and shaded areas indicate ± 1 standard deviation. Class sizes and proportions are shown in the legends.

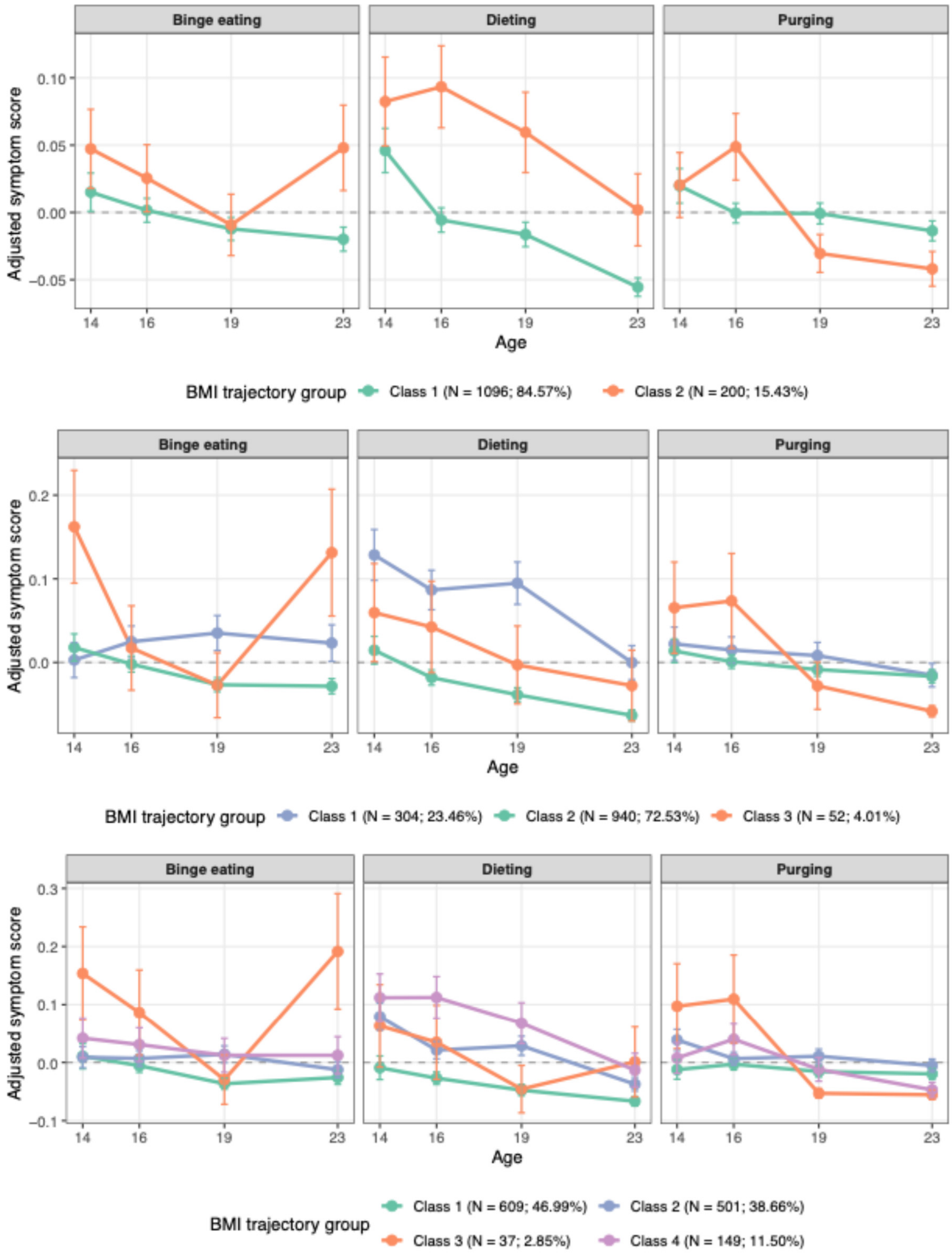

Figure S3. Different longitudinal BMI profile solutions and associated disordered eating behaviours.

Age-related trajectories of dieting, binge eating, and purging symptoms across the corresponding 2-, 3-, and 4-class BMI solutions (top, middle and bottom panels, respectively). Symptom scores were residualised for sex and recruitment site before analysis.

Points represent estimated mean residual scores at each age (14, 16, 19, and 23 years), and error bars indicate standard errors. Across the 3- and 4-class solutions, the additional classes primarily subdivided the elevated BMI profile and showed largely overlapping behavioural profiles, whereas the 2-class solution captured the principal distinction between persistently elevated and normal BMI profiles.

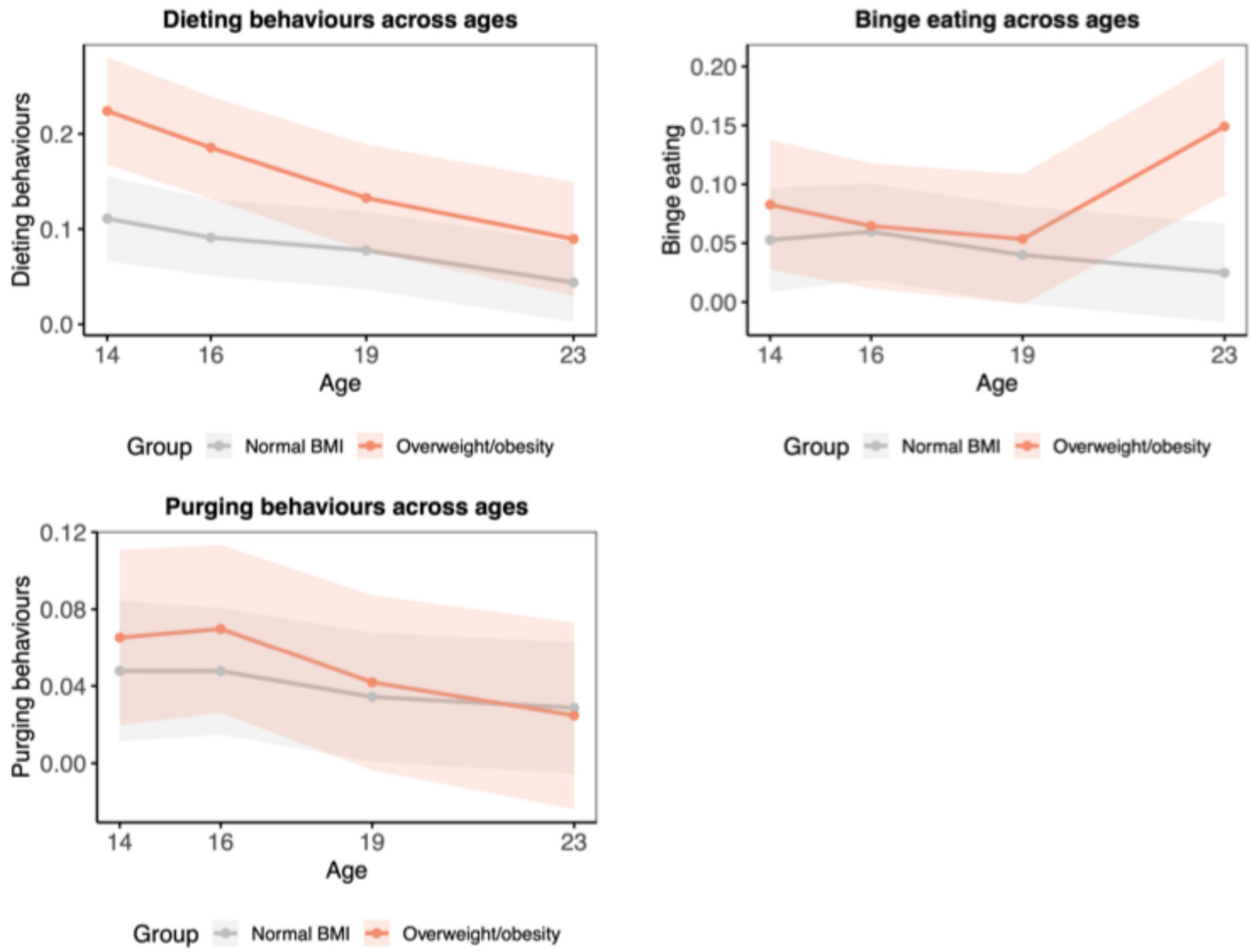

Figure S4: Trajectories of disordered eating behaviours from ages 14 to 23 in participants classified according to baseline BMI status.

Predicted mean levels of dieting (top left), binge eating (top right), and purging behaviours (bottom left) are shown for adolescents with normal BMI (5th- <85th percentile) and overweight/obesity (≥85th percentile) at age 14, based on CDC age- and sex-specific BMI percentiles. Solid lines represent estimated marginal means from mixed-effects models, and shaded areas indicate 95% confidence intervals.

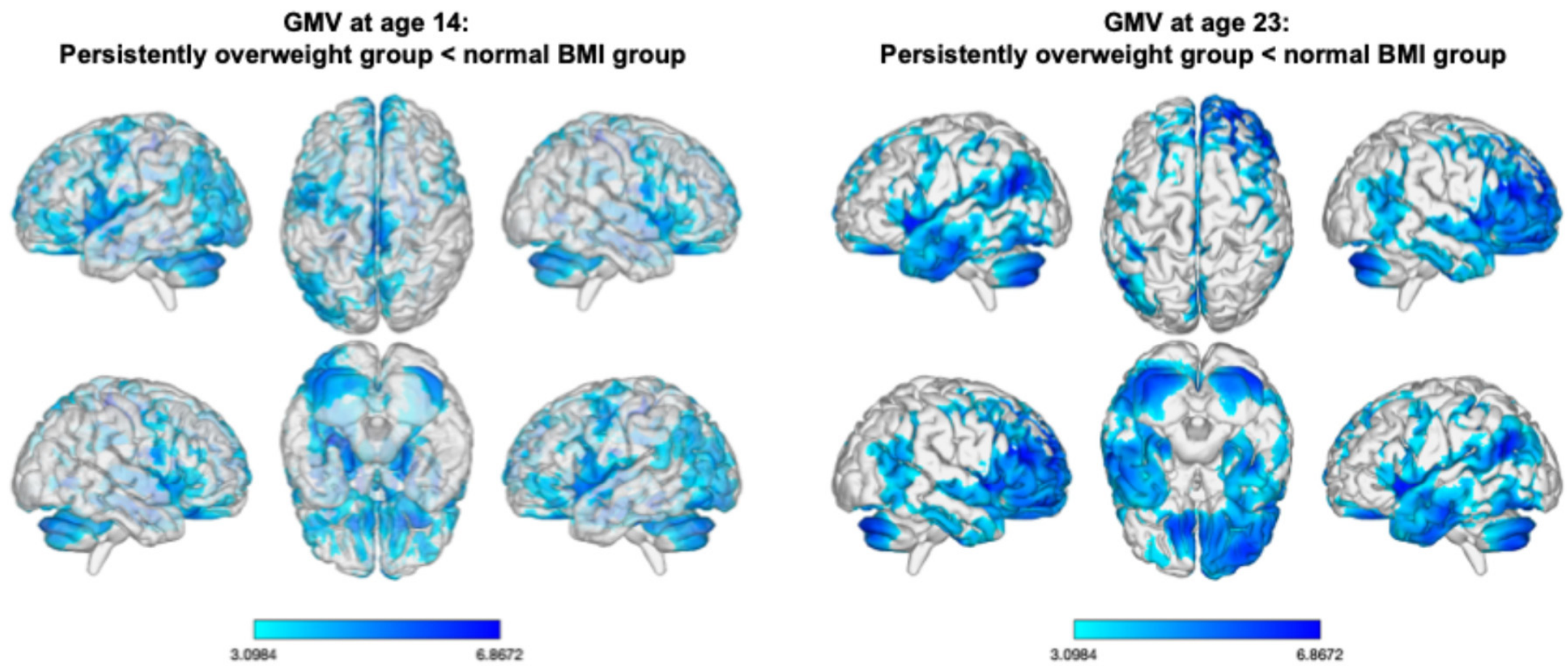

Figure S5. Cross-sectional GMV differences between the persistently high and normal BMI groups.

The colour bar represents T values. For the cross-sectional analyses at ages 14 and 23, total intracranial volume (TIV) at the respective time point was included as a covariate.

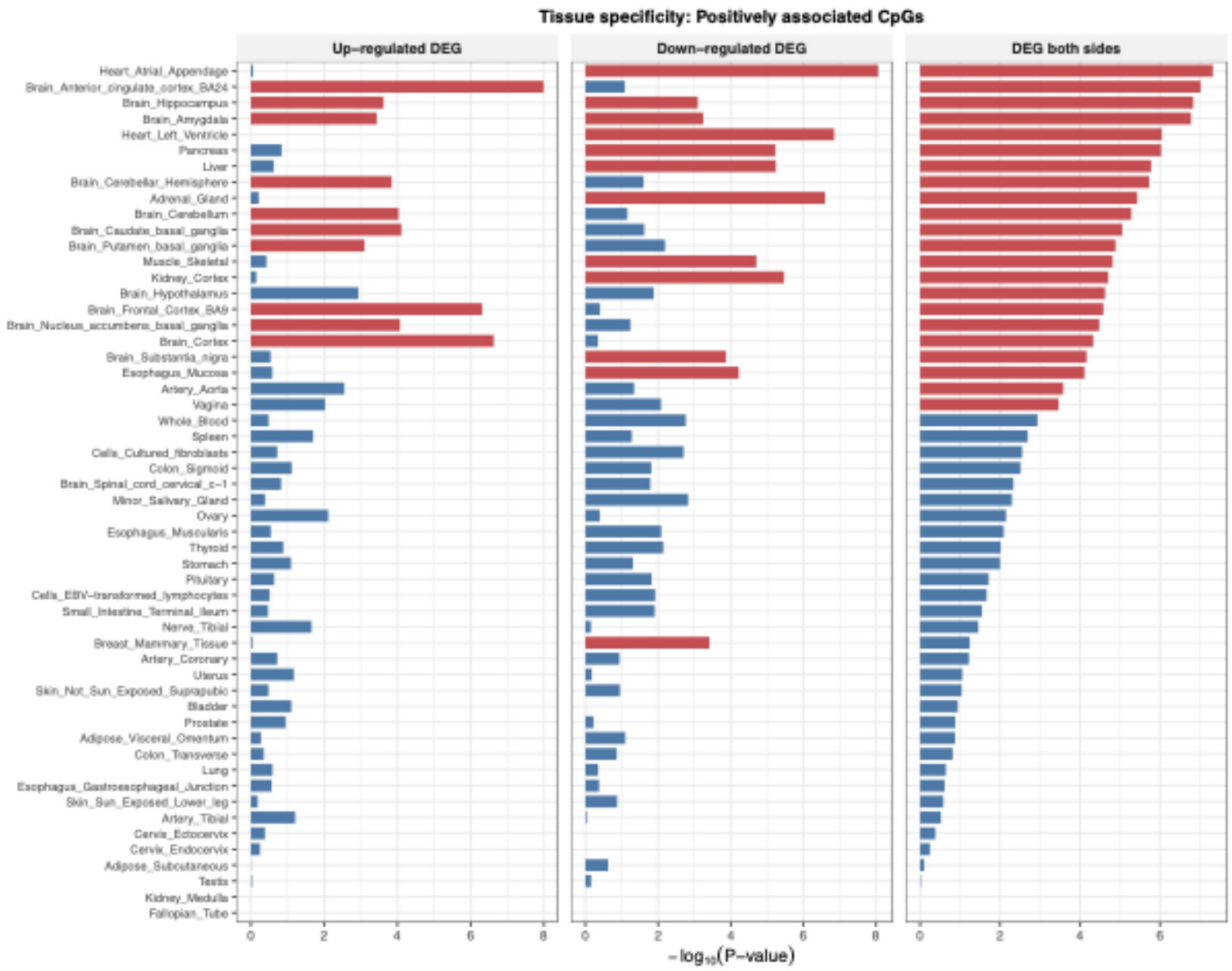
Tissue specificity: Positively associated CpGs
Up−regulated DEG
Down−regulated DEG
DEG both sides
−log10(P−value)

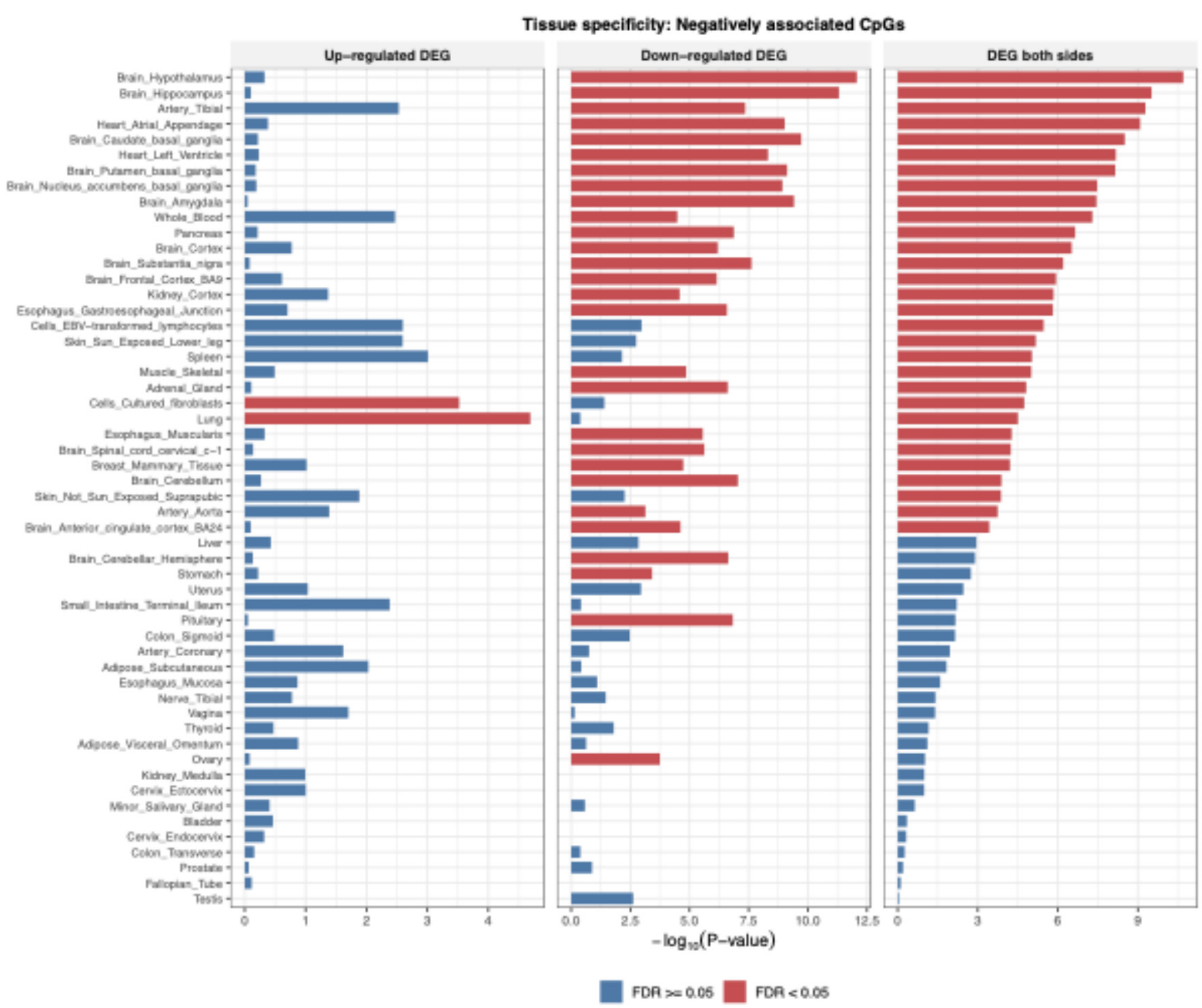
Tissue specificity: Negatively associated CpGs
Up−regulated DEG
Down−regulated DEG
DEG both sides
−log10(P−value)
FDR >= 0.05
FDR < 0.05

Figure S6. Tissue-specific enrichment of all FDR-significant CpGs associated with BMI. Genes nearest to the CpGs positively (top panel) or negatively (bottom panel) associated with BMI were tested for their enrichment for genes known to be differentially expressed in each tissue using GENE2FUNC from the FUMA web application (16). All genes available in FUMA (N = 57,241) were used as the background gene set for enrichment testing. Significantly enriched differentially expressed (DEG) sets (Bonferroni-corrected p-values < 0.05) are highlighted in red. For the graphs representing up-regulated and down-regulated DEG, the direction of expression was considered (i.e., an up-regulated DEG set contains all genes that are overexpressed in each tissue compared to the other samples). The -log10 p-values refer to the probability of the hypergeometric test.

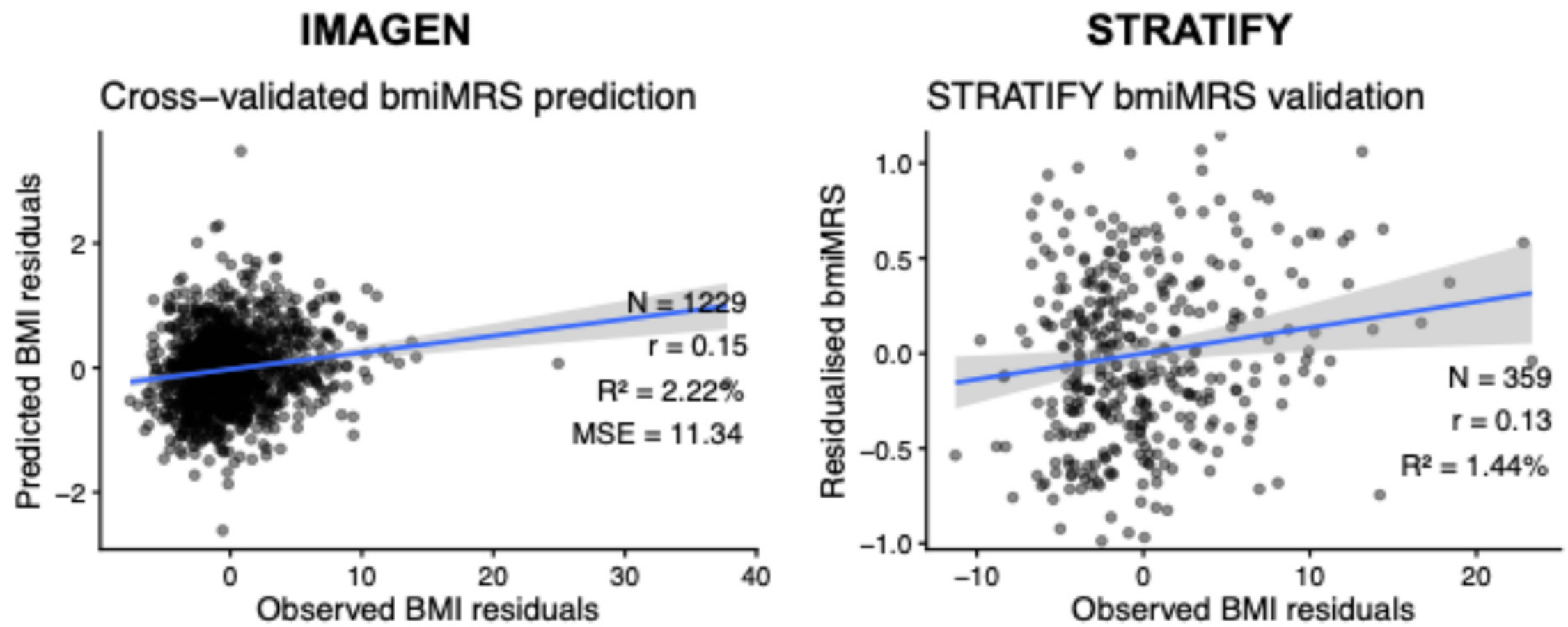

Figure S7. Cross-validation and external validation of the methylation risk score for BMI (bmiMRS).

The bmiMRS was derived in IMAGEN using LASSO penalised regression with ten-fold cross-validation. Left: Association between observed BMI residuals and cross-validated predicted BMI residuals in IMAGEN (*N* = 1,229). Right: Association between residualised bmiMRS and residualised BMI in the independent STRATIFY cohort (*N* = 359). The bmiMRS explained 2.22% of the variance in BMI in IMAGEN and 1.44% of the variance in BMI in STRATIFY. Solid lines represent linear regression fits, and shaded areas indicate 95% confidence intervals.

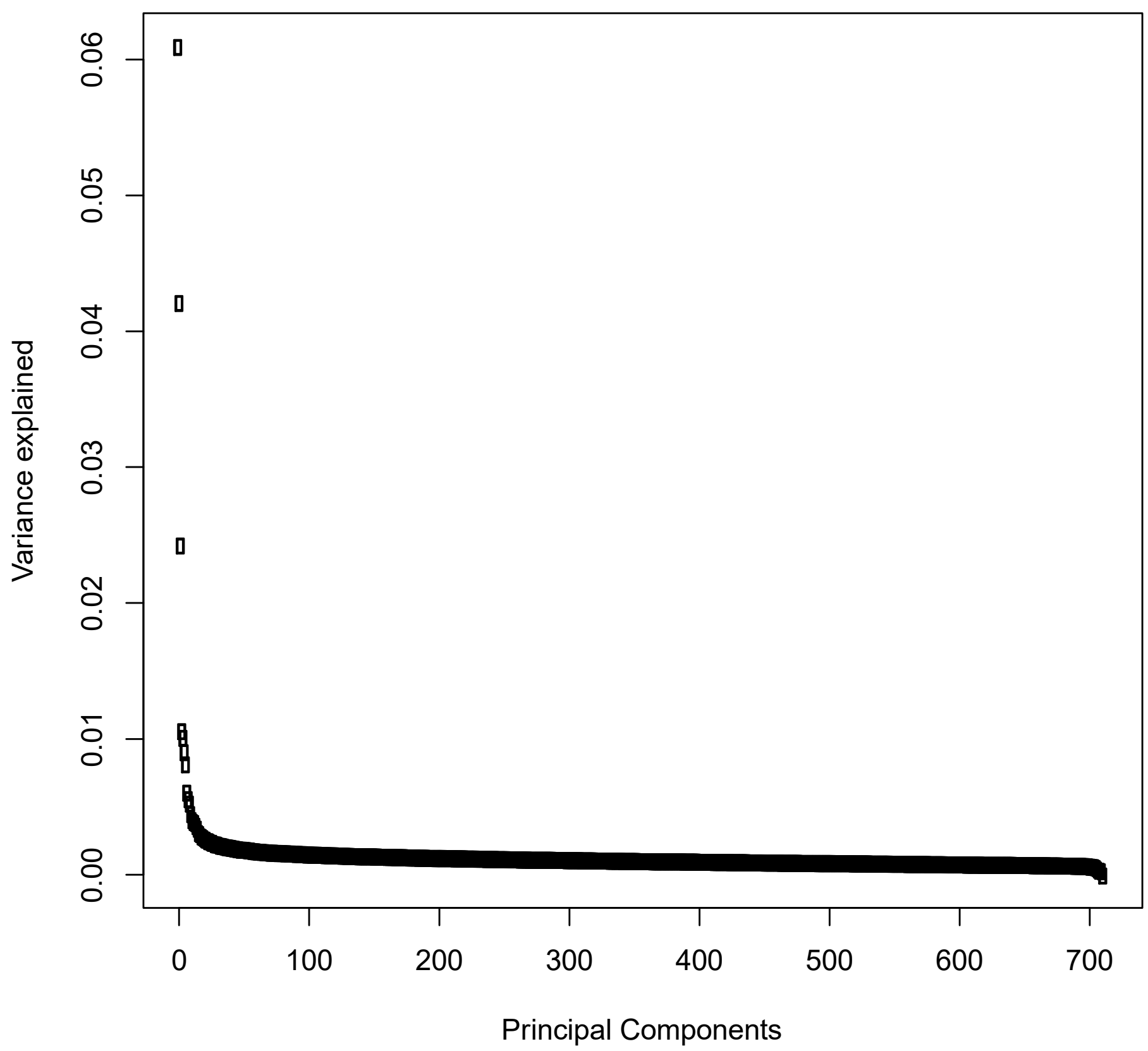

Figure S8. Principal component analysis (PCA) of DNA methylation levels across all autosomal probes.

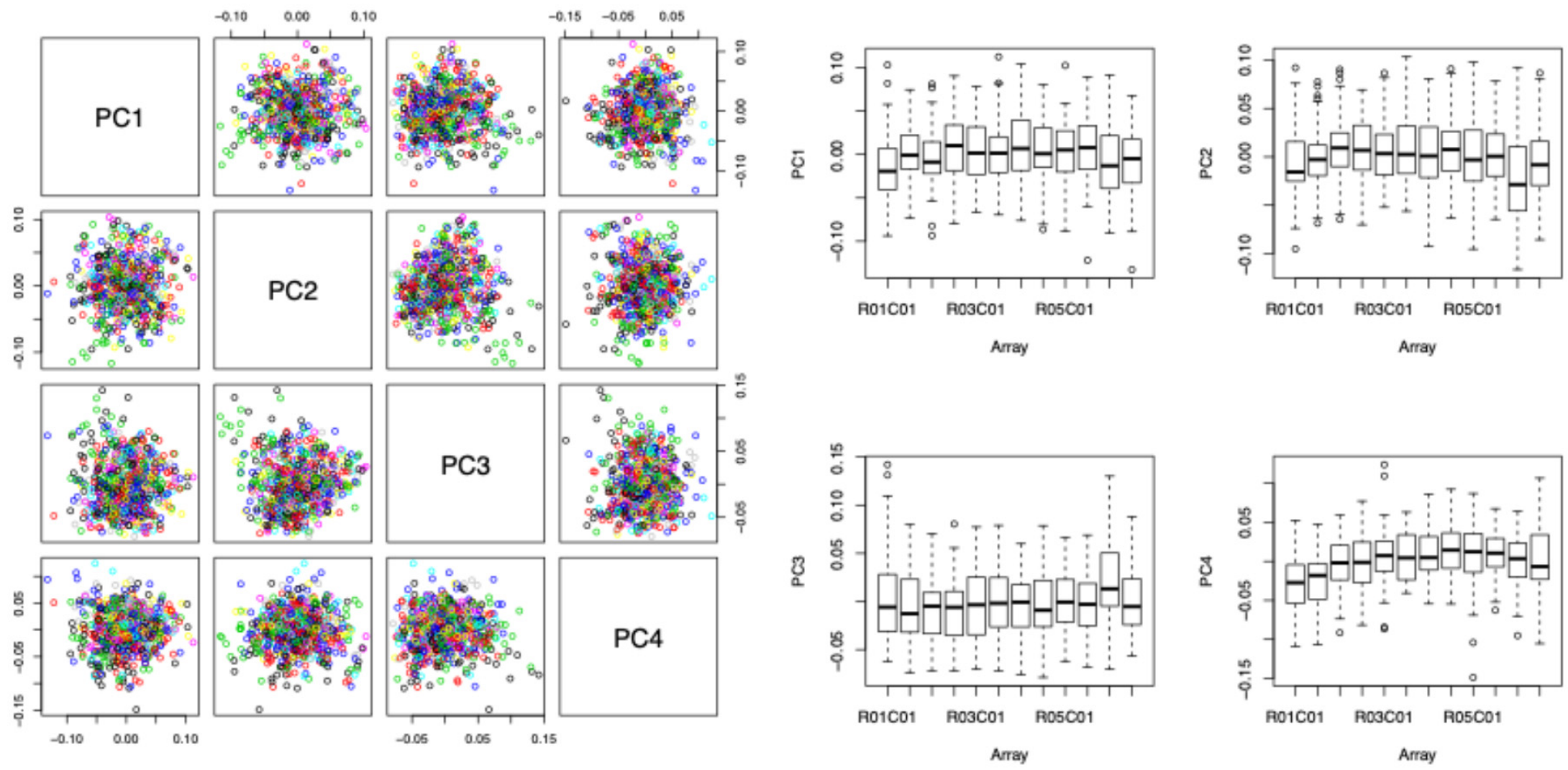

Figure S9. Effects of array location on DNA methylation.

The left panel shows scatterplots of methylation levels across the first four PCs by probe location on the array (colour-coded). The right panel displays the estimated main effects of array position on these PCs. No significant positional effects were observed.

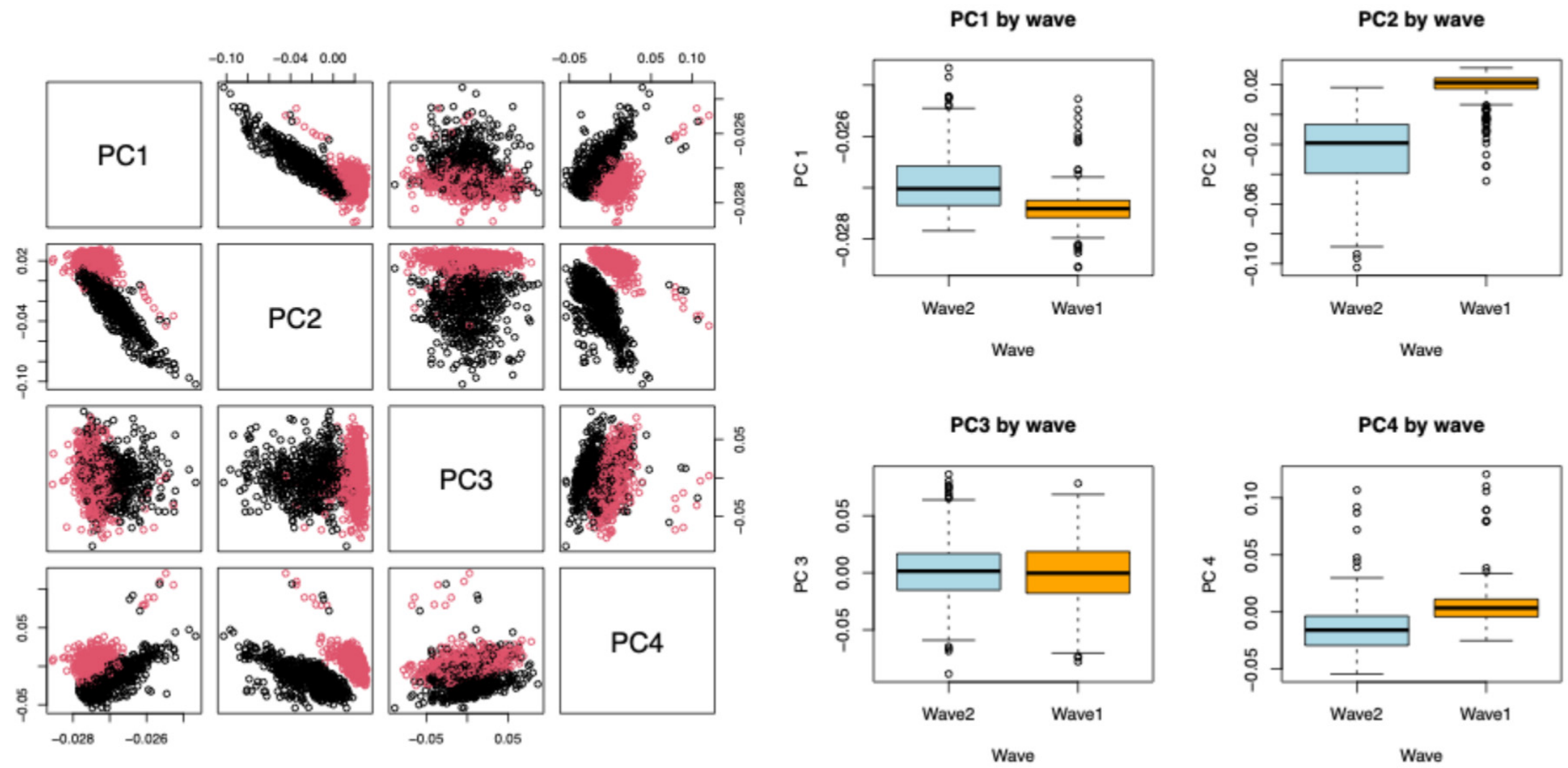

Figure S10. Effects of different processing waves on DNA methylation.

The left panel shows scatterplots of methylation levels across the first four PCs, with samples coloured by processing waves: red for wave 1 and black for wave 2 in black. The right panel presents the distributions of PC1-PC4 between waves 1 and 2. Some differences between waves are observed, indicating wave as a potential confounder; therefore, wave was included as a covariate in all downstream analyses.

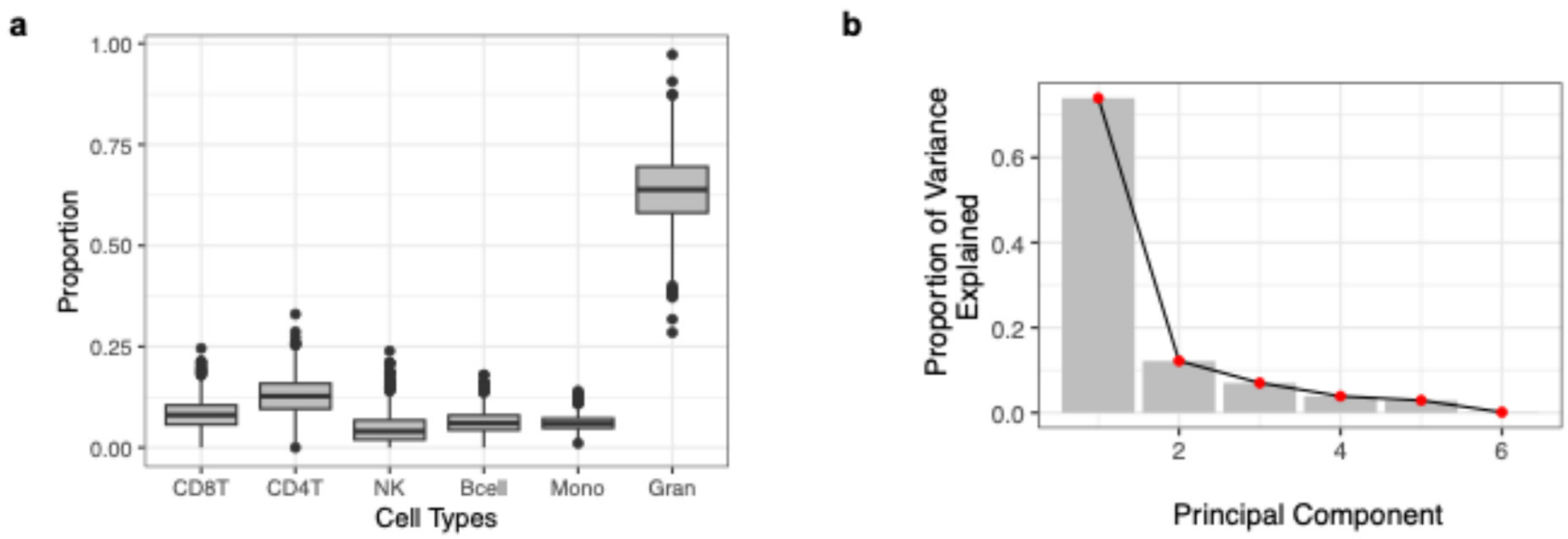

Figure S11. Estimated blood cell-type proportion.

a, Proportions of six major cell types (granulocytes, B cells, CD4+ T cells, CD8+ T cells, monocytes, and natural killer (NK) cells. b, Variance explained by the first six PCs of estimated cell-type proportions.

Supplementary References

1. Goodman R, Ford T, Richards H, Gatward R, Meltzer H (2000): The development and well-being assessment: description and initial validation of an integrated assessment of child and adolescent psychopathology. *J Child Psychol Psychiatry* 41: 645–655.
2. Heatherton TF, Kozlowski LT, Frecker RC, Fagerström KO (1991): The Fagerström Test for Nicotine Dependence: a revision of the Fagerström Tolerance Questionnaire. *Br J Addict* 86: 1119–1127.
3. Newcomb MD, Huba GJ, Bentler PM (1981): A multidimensional assessment of stressful life events among adolescents: Derivation and correlates. *J Health Soc Behav* 22: 400–415.
4. Sun Y, Jia T, Barker ED, Chen D, Zhang Z, Xu J, *et al.* (2022): Associations of DNA methylation with behavioral problems, grey matter volumes and negative life events across adolescence: Evidence from the longitudinal IMAGEN study. *Biol Psychiatry* 93: 342–351.
5. Weller BE, Bowen NK, Faubert SJ (2020): Latent Class Analysis: A Guide to Best Practice. *J Black Psychol* 46: 287–311.
6. Muthén B, Muthén LK (2000): Integrating Person-Centered and Variable-Centered Analyses: Growth Mixture Modeling With Latent Trajectory Classes. *Alcohol Clin Exp Res* 24: 882–891.
7. Ashburner J (2007): A fast diffeomorphic image registration algorithm. *NeuroImage* 38: 95–113.
8. Robert GH, Luo Q, Yu T, Chu C, Ing A, Jia T, *et al.* (2020): Association of Gray Matter and Personality Development With Increased Drunkenness Frequency During Adolescence. *JAMA Psychiatry* 77: 409–419.
9. Esteban O, Birman D, Schaer M, Koyejo OO, Poldrack RA, Gorgolewski KJ (2017): MRIQC: Advancing the automatic prediction of image quality in MRI from unseen sites. *PLOS ONE* 12: e0184661.
10. Aryee MJ, Jaffe AE, Corrada-Bravo H, Ladd-Acosta C, Feinberg AP, Hansen KD, Irizarry RA (2014): Minfi: a flexible and comprehensive Bioconductor package for the analysis of Infinium DNA methylation microarrays. *Bioinforma Oxf Engl* 30: 1363–1369.

11. Chen Y, Lemire M, Choufani S, Butcher DT, Grafodatskaya D, Zanke BW, *et al.* (2013): Discovery of cross-reactive probes and polymorphic CpGs in the Illumina Infinium HumanMethylation450 microarray. *Epigenetics* 8: 203–209.
12. Meng H, Joyce AR, Adkins DE, Basu P, Jia Y, Li G, *et al.* (2010): A statistical method for excluding non-variable CpG sites in high-throughput DNA methylation profiling. *BMC Bioinformatics* 11: 227.
13. Yu X, Zhang Z, Herle M, Banaschewski T, Barker GJ, Bokde ALW, *et al.* (2025): Relationships of eating behaviors with psychopathology, brain maturation and genetic risk for obesity in an adolescent cohort study. *Nat Ment Health* 3: 58–70.
14. Manichaikul A, Mychaleckyj JC, Rich SS, Daly K, Sale M, Chen W-M (2010): Robust relationship inference in genome-wide association studies. *Bioinforma Oxf Engl* 26: 2867–2873.
15. Conomos MP, Miller MB, Thornton TA (2015): Robust inference of population structure for ancestry prediction and correction of stratification in the presence of relatedness. *Genet Epidemiol* 39: 276–293.
16. Watanabe K, Taskesen E, van Bochoven A, Posthuma D (2017): Functional mapping and annotation of genetic associations with FUMA [no. 1]. *Nat Commun* 8: 1826.
